# Speciation along a latitudinal gradient: The origin of the Neotropical cycad sister pair *Dioon sonorense*–*D. vovidesii* (Zamiaceae)

**DOI:** 10.1002/ece3.7545

**Published:** 2021-05-01

**Authors:** José Said Gutiérrez‐Ortega, Francisco Molina‐Freaner, José F. Martínez, Miguel Angel Pérez‐Farrera, Andrew P. Vovides, Antonio Hernández‐López, Ayumi Tezuka, Atsushi J. Nagano, Yasuyuki Watano, Yuma Takahashi, Masashi Murakami, Tadashi Kajita

**Affiliations:** ^1^ Department of Biology Faculty of Science Chiba University Chiba Japan; ^2^ Departamento de Ecología de la Biodiversidad Instituto de Ecología Universidad Nacional Autónoma de México Hermosillo Mexico; ^3^ Laboratorio de Ecología Evolutiva Herbario Eizi Matuda Instituto de Ciencias Biológicas Universidad de Ciencias y Artes de Chiapas Tuxtla Gutiérrez Mexico; ^4^ Departamento de Biología Evolutiva Instituto de Ecología Xalapa Mexico; ^5^ Ciencias Agrogenómicas Escuela Nacional de Estudios Superiores Universidad Nacional Autónoma de México León Mexico; ^6^ Faculty of Agriculture Ryukoku University Otsu Japan; ^7^ Iriomote Station Tropical Biosphere Research Center University of the Ryukyus Yaeyama Japan; ^8^ United Graduate School of Agricultural Science Kagoshima University Kagoshima Japan

**Keywords:** aridification, cycads, demographic history, environmental gradient, geographic range margins, niche conservatism

## Abstract

Latitude is correlated with environmental components that determine the distribution of biodiversity. In combination with geographic factors, latitude‐associated environmental variables are expected to influence speciation, but empirical evidence on how those factors interplay is scarce. We evaluated the genetic and environmental variation among populations in the pair of sister species *Dioon sonorense–D. vovidesii*, two cycads distributed along a latitudinal environmental gradient in northwestern Mexico, to reveal their demographic histories and the environmental factors involved in their divergence. Using genome‐wide loci data, we determined the species delimitation, estimated the gene flow, and compared multiple demographic scenarios of divergence. Also, we estimated the variation of climatic variables among populations and used ecological niche models to test niche overlap between species. The effect of geographic and environmental variables on the genetic variation among populations was evaluated using linear models. Our results suggest the existence of a widespread ancestral population that split into the two species ~829 ky ago. The geographic delimitation along the environmental gradient occurs in the absence of major geographic barriers, near the 28th parallel north, where a zonation of environmental seasonality exists. The northern species, *D. vovidesii*, occurs in more seasonal environments but retains the same niche of the southern species, *D. sonorense*. The genetic variation throughout populations cannot be solely explained by stochastic processes; the latitudinal‐associated seasonality has been an additive factor that strengthened the species divergence. This study represents an example of how speciation can be achieved by the effect of the latitude‐associated factors on the genetic divergence among populations.

## Graphical abstract

We reconstructed the history of lineage divergence that gave origin to the sister cycad species *Dioon* *sonorense*–*D. vovidesii* from northwestern Mexico. We found that the two lineages split approximately 1 million years ago, and their current geographic separation is concordant with the environmental seasonality variation associated with the latitudinal environmental gradient that, in turn, determines the margin of the Neotropical forests. The divergence that led to speciation was promoted by (a) a geographic component that separated the two neighboring lineages, and (b) an environmental component that increased the distinction between them.

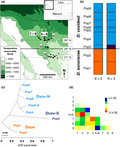

## 1 INTRODUCTION

Latitudinal variation comprises a combination of geographic and environmental components that may determine the abundance and the limits of the distribution of biological groups (Janzen, 1967; Willig et al., 2003). The effect of the latitude‐associated environmental factors on biodiversity has been studied from diverse perspectives, including community ecology (Stevens, 1989), biogeography (Mittelbach et al., 2007), or evolutionary biology (Van Dievel et al., 2019). These different fields of study concordantly suggest that biotic and abiotic factors that covary in association with latitude can influence adaptation or demographic dynamics, which may facilitate evolutionary divergence among neighboring communities or populations distributed at different positions in latitude (Ghalambor et al., 2006). This idea implies that latitudinal variation among populations might be also important in speciation (Kozak & Wiens, 2007).

Since latitudinal environmental gradients provide zonations that can influence contrasting evolutionary trends in neighboring lineages (Hua & Wiens, 2010; Kozak & Wiens, 2007), they seem to be ideal scenes for the processes of lineage divergence and speciation (Mayr, 1954). However, there is scarce empirical evidence on how environmental and geographic factors interplay to promote speciation in these geographic configurations. In general, genetic drift is expected to be stronger in populations at higher latitudes than in their low‐latitude counterparts due to drastic demographic changes such as population fluctuations or founder effect due to climatic factors (e.g., glaciations) (Cortázar‐Chinarro et al., 2017). Likewise, natural selection regimes can differ at local and regional scales among populations because ecological conditions can vary with latitude (Takahashi et al., 2014). Besides, gene flow may contribute to reducing differentiation among populations, counteracting any divergence (Nosil, 2008). To identify the factors involved in speciation occurring along latitudinal environmental gradients, it is necessary to reveal the relative contribution of ecological and demographic factors involved in the origin and maintenance of local alleles and population divergence (Wiens, 2004) and understand how such factors vary in relation with latitude.

**FIGURE 1:**
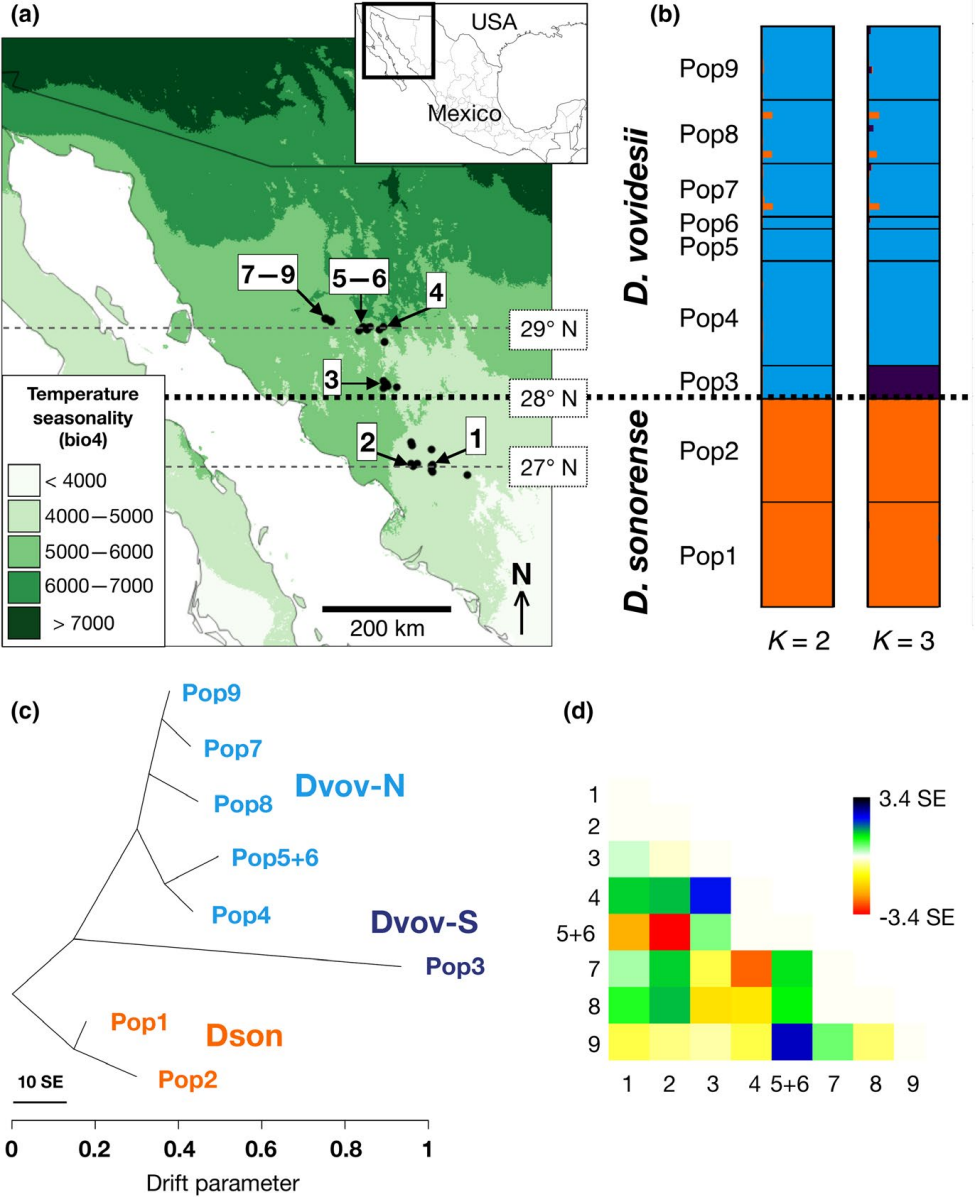
Species delimitation between *Dioon sonorense* and *D. vovidesii*. (a) Geographic distribution of populations of *D. sonorense* (Pops 1–2) and *D. vovidesii* (Pops 3–9) covered in this study. Population numbers correspond to those indicated in Table 1 and Table S1. Dotted lines indicate the 28th parallel north, which roughly represents the northernmost limit of the Neotropical forests in western Mexico (Martínez‐Yrízar et al., 2000). The green‐scaled color indicates the geographic variation of temperature seasonality (standard deviation of annual mean temperature × 100) according to the bio4 raster layer of WorldClim (Hijmans et al., 2005; http://www.worldclim.org). (b) Genetic clusters were estimated with 809 neutral loci from nine populations in STRUCTURE (Pritchard et al., 2010). Clustering *K* = 2 clearly delimitates the two species, and *K* = 3 separates the set in three lineages: Dson, Dvov‐S, and Dvov‐N. More detailed information of the STRUCTURE results when *K* = 2–10 can be found in Figure S2. (c) The best maximum‐likelihood graph estimated in TreeMix (Pickrell & Pritchard,^2012^) using 809 neutral SNPs contains no migration edges. Horizontal branch lengths are proportional to the amount of genetic drift. The scale bar shows 10 times the *SE* in the sample covariance matrix. (d) Covariance matrix among populations, showing −3.4–3.4 *SE*

The cycad genus *Dioon* comprises 17 species with allopatric or parapatric distribution ranges, scattered throughout the northernmost Neotropical provinces of Mexico and Honduras (Calonje et al., 2021; Gutiérrez‐Ortega, Salinas‐Rodríguez, et al., 2018). Whereas the only Honduran species represents the southern distribution margin of the genus, the northernmost margin occurs in the State of Sonora, Mexico. In this northern limit, the sister pair *D. sonorense* (De Luca, Sabato & Vázq. Torres) Chemnick, T.J. Greg. & Salas‐Mor. and *D. vovidesii* Gutiérrez‐Ortega & Pérez‐Farr. co‐occur in parapatry between the parallels *ca*. 27 and 29° N (Figure 1a). Previously, the populations of *D. sonorense* and *D. vovidesii* were considered to be part of the same species (the former concept of *D. sonorense*). In recent studies, we demonstrated that *D. sonorense* actually consists of two very closely related species that can be distinguished using either genetic, morphological, or leaflet anatomical analyses (Gutiérrez‐Ortega, Kajita, Molina‐Freaner, 2014; Gutiérrez‐Ortega, Jiménez‐Cedillo, Pérez‐Farrera, Martínez, et al., 2018; Gutiérrez‐Ortega, Jiménez‐Cedillo, Pérez‐Farrera, Vovides, et al., 2018) *D. sonorense* comprises the cycad populations in southern Sonora and northern Sinaloa; and *D. vovidesii* comprises the populations from central Sonora. There are no conspicuous geographic barriers to distinguish the spatial separation between *D. sonorense* and *D. vovidesii*, but the habitats for both species are clearly different: *D*. *sonorense* inhabits seasonally dry forests of Neotropical origin (Álvarez‐Yépiz et al., 2011), whereas *D. vovidesii* lives in areas with abounding xerophytic scrublands or *Quercus* forests, with closer affinities with the Nearctic flora (Rzedowski & Huerta, 1978). Previous floristic studies in Sonora have suggested that the limit of the Neotropical forests coincides with the 28th parallel north (Martínez‐Yrízar et al., 2000), and this is well noticed when considering climatic variables associated with seasonality (Figure 1a). Although the marginal populations of each species are easy to distinguish, there are still doubts regarding the taxonomic status of an intermediate population near the 28th parallel north (Pop3 in Figure 1a). Pop3 shares the haplotype of the trn*L‐F* region of the chloroplast DNA of populations of *D. vovidesii* (Gutiérrez‐Ortega, et al., 2014) (Table 1), but the leaf morphology of plants differs with respect to the two species, with anomalously longer and twisted leaves (rachis), narrower and more separated leaflets, and with obtuse angles of leaflet insertion. In a previous study, we suggested this population represents an outlier because it has been affected by intensive illegal collection; the population has been decimated and the remaining plants are depauperate (Gutiérrez‐Ortega, et al., 2014). For those reasons, we were unable to add Pop3 in our most recent study (Gutiérrez‐Ortega, Jiménez‐Cedillo, Pérez‐Farrera, Vovides, et al., 2018), but its geographic position is important to understand the delimitation between *D. sonorense* and *D. vovidesii* along the environmental gradient. If the two species truly evolved in different habitats, and if Pop3 truly belongs to *D. vovidesii*, as the variation of the *trnL‐F* region suggests, the species delimitation would coincide with the environmental zonation of seasonality near the 28th parallel north (Figure 1a).

**TABLE 1:** Sampled populations and number of samples of *Dioon sonorense* and *D. vovidesii* examined in this study

Species	Population		Samples	*trnL‐F* Haplotype	Lineage
*Dioon sonorense*	1	Choquincahui	16	B	Dson
	2	Álamos	16	B	Dson
*Dioon vovidesii*	3	Nuri	5	A_i_	Dvov‐S
	4	Bacanora	16	A_i_	Dvov‐N
	5	Bacanora‐El Novillo	5	A	Dvov‐N
	6	El Novillo	2	A	Dvov‐N
	7	SM, Iglesias	8	A	Dvov‐N
	8	SM, Cañada	10	A	Dvov‐N
	9	SM, Cueva	11	A	Dvov‐N

Note

The haplotype of the intergenic region *trnL‐F* of the chloroplast DNA (cpDNA) detected in Gutiérrez‐Ortega et al. (2014) is annotated. Populations correspond to either of the three lineages (Dson, Dvov‐S, or Dvov‐N) determined in this study (see Results). SM = Sierra de Mazatán. Geographic coordinates for all localities are deliberately omitted to discourage illegal collecting.

In this study, we reveal the mechanisms of speciation involved in the history of divergence between *D. sonorense* and *D. vovidesii*. In particular, we aim to (a) clarify the species delimitation; (b) estimate population genetic parameters and reveal the demographic history of lineages; (c) estimate the climatic factors involved in the divergence of the species; and (d) examine the influence of the latitudinal environmental gradient on their genetic diversity and differentiation.

## 2 MATERIALS AND METHODS

### 2.1 Restriction‐associated DNA sequencing

We used 89 individuals from nine populations throughout the distribution range of *D. vovidesii* and two populations of *D. sonorense* (Figure 1; Table 1 and Table S1). Due to their low sampling size, and because they are geographically close, populations 5 and 6 were merged into a single population in all following analyses. Genomic DNA was extracted according to the protocol of Doyle and Doyle (1987) and digested with *Bgl* II and *Eco RI* restriction enzymes. Double‐digest restriction‐associated DNA sequencing (ddRAD‐seq) and raw sequence filtering were performed following the protocol described in Gutiérrez‐Ortega, Salinas‐Rodríguez, et al. (2020).

### 2.2 Constructing and filtering of SNP dataset

The software pipeline Stacks v2.4 (Catchen et al., 2013) was used for the RAD‐seq data filtering and SNP discovery. “Ustacks” was used to assemble a minimum of five intra‐individual reads (*m* = 5) with maximum distance (*M*) = 2, enabling the deleveraging (*d*) and removal (*r*) algorithms. “Cstacks” was used to create a catalog with a maximum of four mismatches between loci (*n* = 4). “Sstacks” was used to search the ustacks products into the catalog produced in “cstacks.” “Populations” was used to retrieve SNPs from polymorphic loci that were present in at least 90% of individuals (*r* = .9). When loci were not detected in a sample, alleles were treated as missing data. Minor alleles with frequency <.05 were excluded. One randomly selected SNP was retrieved from each locus using the option “write_random_snp.” This first screening revealed a total of 1989 loci. Data were exported as a STRUCTURE file format, and all file conversions after this step were done with the programs Formatomatic v0.8.1 (Manoukis, 2007), PGDSpider v2.1.1.1 (Lischer & Excoffier, 2012), or PLINK v1.9 (Purcell et al., 2007). A second filtering screening was done using the Hardy–Weinberg equilibrium (HWE) test in GenAlEx v6.5 (Peakall & Smouse, 2012). A total of 1,118 loci with significant departure from HWE within any population were discarded from our dataset, which gave a dataset consisted of 871 loci.

A third filtering step consisted of distinguishing the outlier loci. The R package fsthet (Flanagan & Jones, 2017) was used to detect loci with *F_st_* values lower or higher than the confidence interval 95. Besides, the Bayesian approach in BayeScan (Foll & Gaggiotti, 2008) was employed using 100,000 iterations, 50,000 burn‐in periods, 100 prior odds, and default values in the rest of the parameters. Using both methods, 62 loci were detected as outliers (Figure S1). This allowed us to manage three datasets, each consisting of all 871 SNPs (total data), 809 neutral loci only, and 62 outlier loci only.

### 2.3 Genetic diversity and structure

For the three datasets, phi‐values of pairwise genetic distances among individuals were calculated in GenAlEx v6.5 (Peakall & Smouse, 2012), enabling the option “interpolate missing data.” The three matrices of phi‐values were used to perform an analysis of molecular variance (AMOVA) using 999 permutations and principal coordinate analyses (PCoA) in GenAlEx. GenAlEx was also used to estimate genetic diversity parameters on the neutral loci dataset. Effective allele richness (*A_e_*), observed (*H_o_*) and expected heterozygosity (*H_e_*), inbreeding coefficient (*F*), and Shannon's information index (*I*) were estimated at the population and the species levels.

The software STRUCTURE 2.3.3 (Pritchard et al., 2010) was used to test the genetic clustering across populations under an admixture model, using the neutral loci dataset. Ten iterations of 100,000 burn‐in periods and 1 million MCMC repetitions were run. One to ten clusters (*K = *1–10) were tested. Optimal *K* values were determined using the Delta‐*K* method of Evanno et al. (2005) in Structure Harvester (Earl & vonHoldt, 2012). Results were visualized using CLUMPAK (Cluster Markov Packager Across K) (Kopelman et al., 2015).

### 2.4 Population splits and admixture

Using the neutral dataset, the software TreeMix v1.13 (Pickrell & Pritchard, 2012) was used to reconstruct the population splits during the divergence of *D. sonorense* and *D. vovidesii*. TreeMix uses allele frequency data under a Gaussian approximation to genetic drift; thus, it produces a maximum‐likelihood tree of population ancestry that allows visualizing the relative effect of genetic drift among populations. We used eight independent runs assuming 0–7 migration events to test whether the addition of migration events improves the model fit to the data. The best tree was selected by comparing the likelihood scores of the eight runs. Also, we used the *f*3 statistic (Reich et al., 2009) implemented in TreeMix to detect evidence of admixture among the three lineages we identified in the genetic structure analyses (see Results).

### 2.5 Gene flow estimation

Historical gene flow was estimated among populations, among three lineages determined in genetic structure analyses (see Results), and between species, using MIGRATE‐N v3.6 (Beerli & Felsenstein, 1999, 2001). In all cases, the pairwise mutation‐scaled migration rates (*M*) over 4*N_e_* generations in the past were estimated under a stepwise mutation model assuming constant mutation rates across loci. *F_st_* values were used as starting parameter to calculate *M*. Each run assumed uniform priors ranging from 0 to 0.25 and delta = 0.025 to estimate theta (*Θ*) and uniform prior distribution between 0 and 2000 with delta = 200 to estimate *M*. Metropolis‐coupled MCMC consisted of one long chain of 25,000 steps, sampling every 20 steps. Burn‐in period was set to 100,000. The numbers of migrants per generation (*N_m_*) were estimated with the formula *Θ_a_M_a_*_‐>_ *_b_* = 4*N_m__a_*.

### 2.6 Inference of demographic history

DIYABC v2.1.0 (Cornuet et al., 2014) was used to test different scenarios that might explain the demographic history of divergence between *D. sonorense* and *D. vovidesii*. This software implements the approximate Bayesian computation (Beaumont et al., 2002), which uses prior information and genetic diversity and pairwise differentiation of populations as summary statistics to estimate posterior probabilities. Mutation rate is assumed to be equal among loci when estimating divergence times (Beaumont et al., 2002). The most likely scenario is determined by comparing the posterior probabilities values with the observed values.

For this analysis, we first used the 809 neutral SNP datasets. However, despite using alternative scenarios and priors, convergence was not reached in the estimation of all parameters. This was likely due to low informative summary statistics of the 809 SNP datasets (Burr & Skurikhin, 2013). Thus, we needed to build a dataset that considered a more even number of samples per population and a higher number of SNPs. To build this subset, we used the “Ustacks" data of 40 samples representing the five samples per population with the lowest proportion of missing data, as estimated in the previous dataset preparation. We created a new catalog using the same options for “cstacks” and “sstacks” mentioned above. The program “populations” was used to retrieve one SNP per locus, allowing no missing data (*p* = 1). These options gave a dataset of 4,630 SNPs that were used as the input file in DIYABC. However, in the DIYABC input file, we specified to exclude SNPs with minor allele frequency <.05, and the run used the information of a final dataset consisting of 1,138 SNPs.

Four demographic scenarios were tested, all assuming *D. sonorense* and *D. vovidesii* as sister species. The three population groups revealed in Structure and PCoA (Dson, Dvov‐S, Dvov‐N; see Results) were considered as different lineages; the effective population sizes of these three lineages were equal to *N1*, *N2,* and *N3* (respectively). Two scenarios assumed that *N2* was originated by secondary contact between *N1* and *N3*.

- Scenario 1: The ancestral lineage of *D. vovidesii* (*Nc*) diverged from the ancestral lineage of *D. sonorense* (*Na*) *t2* generations ago; *N2* derived from *Nc t1* generations ago; the three lineages had a change in effective population size *t1* generations ago.
- Scenario 2: The ancestral lineage of *D. sonorense* (*Na*) diverged from the ancestral lineage of *D. vovidesii* (*Nc*) *t2* generations ago; *N2* derived from *Nc t1* generations ago; the three lineages had a change in effective population size *t1* generations ago.
- Scenario 3: The ancestral lineage of *D. vovidesii* (*Nc*) diverged from the ancestral lineage of *D. sonorense* (*Na*) *t2* generations ago; *N2* is the result of an admixture event between *Na* and *Nc* t1 generations ago. *Na* and *Nc* had a change in effective population size *t1* generations ago.
- Scenario 4: The ancestral lineage of *D. sonorense* (*Na*) diverged from the ancestral lineage of *D. vovidesii* (*Nc*) *t2* generations ago; *N2* is the result of an admixture event between *Na* and *Nc* t1 generations ago. *Na* and *Nc* had a change in effective population size *t1* generations ago.

As prior information, the *Θ* values of Dson and Dvov‐N estimated in MIGRATE‐N were used to roughly calculate *N1 and N3* via *Θ* = 4*µ* *N_e_*, considering *µ* = 6.70325E−10 substitutions per site/year as estimated for Cycadales by De La Torre et al. (2017), and assuming generation times of 100 years as estimated by Vovides (1990). Such estimations were *N1* = 36,997 and *N3* = 49,416. These values were assumed to be the mean of a normal prior distribution with a standard deviation of 10,000, with 10–100,000 as limits of the distribution. We assigned a uniform prior distribution to the admixture parameter *ra*, with minimum and maximum limits of .001 and .999. For the rest of the parameters, we assigned uniform prior distributions with limits 10 and 100,000 for the ancestral effective population sizes (*Na* and *Nc*) and 10–50,000 for Dvov‐S, *t1*, and *t2*. We assumed that *N1* > *N2* < *N3*, *t2* => *t1*, *Na* <= *N1*, *Nc* <= *N3*. The levels of genetic diversity of the three lineages, *F_st,_* and Nei distances among lineages were used as summary statistics. The run consisted of 8 million simulations.

The most likely scenario was chosen by comparing the posterior probabilities through a logistic regression test (Beaumont et al., 2002; Cornuet et al., 2014). The confidence of the chosen scenario was estimated by calculating the posterior predictive error over 1,000 datasets. Parameters were estimated with a logistic regression for the best‐supported scenario using the 1% stimulated data closest to the observed data.

### 2.7 Analyses of environmental variation

We obtained geographic coordinates of *D. sonorense* and *D. vovidesii* populations from two sources: (a) information of herbaria specimens deposited in MEXU, USON, and HEM; this includes specimens that we deposited after fieldwork of this study; and (b) the Global Biodiversity Information Facility (GBIF, https://www.gbif.org). Our dataset gathered a total of 23 occurrence points, nine of *D. sonorense* and 14 of *D. vovidesii*. We used the software QGIS (http://qgis.osgeo.org) to retrieve environmental data from the 19 bioclimatic layers of resolution of 1 km^2^ deposited in WorldClim (Hijmans et al., 2005) (http://www.worldclim.org/) and the Mexican layer of altitude deposited in INEGI (http://www.inegi.org.mx/geo/contenidos/datosrelieve/continental/continuoelevaciones.aspx). This dataset was used to test the differentiation of variables between *D. sonorense* and *D. vovidesii* through Welch's ANOVA and the Kruskal–Wallis test implemented in PAST v3.14 (Hammer et al.,  2001).

The vector layer of physiographic regions of Mexico (Cervantes‐Zamora et al., 1990) was used to draw a polygon that included the regions where *D. vovidesii*, *D. sonorense,* and the closely related species *D. tomasellii* occur (Table S2). The 20 environmental layers mentioned above were clipped into the extension of these regions. Then, the pairwise correlation between variables in this area was estimated with the program ENMTOOLS (Warren et al., 2010). Seven variables that showed correlations *r* > 0.9 or <−0.9 were removed (Table S3). Twelve noncorrelated layers were retained after this analysis, and a principal component analysis (PCA) of the climate data of the 23 occurrence points was performed. The values of the two main principal components (PCs) were interpolated by generation a continuous surface by simple kriging interpolation (Oliver & Webster, 1990) using 1 s of resolution and default options in QGIS.

Ecological niche models (ENMs) were constructed with MaxEnt v3.3.1 (Phillips et al., 2006) for the two species separately. The set of 12 noncorrelated variables was used, and the option “jackknife to measure variable importance” was enabled to estimate the relative contribution of variables. Runs consisted of 25 bootstraps. The prediction capabilities of the models were evaluated by calculating the areas under the curve (AUC) of the receiver operating characteristic (Phillips et al., 2006).

The obtained ENM average layers were used in ENMTOOLS (Warren et al., 2010) for the following analyses. Niche breadth of the species was estimated with the B1 (inverse concentration) metric. The empirical niche overlap between ENMs was estimated with the *I* index (Warren et al., 2008), which consists of values between 0 (no overlap) and 1 (full overlap). The background test was done to test whether the niches of *D. sonorense* and *D. vovidesii* were similar or more conserved or diverged than expected by chance. In this test, we estimated the overlap between the ENM of *D. sonorense* and 100 replicate ENMs constructed from random points sampled from the background of *D. vovidesii* and vice versa. The null hypothesis of niche similarity cannot be rejected if the background of both species can predict by randomness the niche of the opposite species. This can be confirmed if the empirical overlap value (the *I* index) falls within the 95th percentile of the expected distribution (*p* < .05). Niche divergence or niche conservatism can be confirmed if the empirical overlap is lower or higher (respectively) than that estimated for the ENM of any species and the background of the opposite species (i.e., if the observed value falls outside the 95th percentile of any of the two null distributions; *p* < .05) (McCormack et al., 2010; Warren et al., 2008, 2010). The background of each species was defined as the buffer area within a radius of 100 km around the location of populations.

### 2.8 Effect of the latitudinal environmental gradient on the genetic variation

We first estimated Z‐scores of the genetic diversity values (*A_e_*, *H_o_*, and *Θ*) of the nine populations and estimated their correlation with latitude and the PC1 and PC2 scores in R 3.5.1 (R Core Team, 2020). Also, we used correlation and linear regression tests to identify whether divergence among populations of the two cycad species can be better explained by the latitudinal differences, the effect of geographic isolation, or ecological dissimilarities. For the three genetic differentiation datasets (“neutral,” “all loci,” and “outliers”), we calculated the genetic distances by estimating the phi‐values with 999 permutations in GenAlEx (Peakall & Smouse, 2012). Linear geographic distances among populations were estimated from the geographic coordinates of each population in GenAlEx. Environmental dissimilarities were estimated by calculating the Euclidean distances among populations with the software PAST v3.14( Hammer et al.,  2001), using the 12 noncorrelated layers dataset. Because the climatic variable bio4 (temperature seasonality) was found to be important on the distinction between species' environments (see Results), we treated it separately, and the dissimilarity among populations was also estimated with PAST. Latitude differentiation between populations was calculated manually. These datasets are shown in Table S4.

Using simple Mantel correlations with the R package “vegan” (Oksanen et al., 2017) with 999 permutations, we confirmed that the latitudinal differentiation, environmental dissimilarities, and geographic distances among populations are all correlated (Table S5). Thus, we needed to distinguish the relative effect of each variable on genetic differentiation. For that, we constructed multiple linear regression models in R 3.5.1 (R Core Team, 2020), in which the genetic differentiation among populations was considered as a dependent variable, whereas geographic distances, environmental dissimilarity, latitudinal differentiation, the PC1 scores, and temperature seasonality (bio4), or their interactions were considered independent variables. For each genetic differentiation dataset (“neutral,” “all loci,” and “outliers”), we built 37 models representing all possible combinations and interactions among the independent variables. The fitness of the data in the models was assessed using the Akaike information criterion corrected for small samples (AICc) with the R package MuMIn (Bartón, 2020).

### 2.9 Identification of natural selection regimes on population groups

We used the ratio between the phi‐value of outlier loci and neutral loci (Phi_(outlier)_/Phi_(neutral)_) to infer differences in the local natural selection regimes among populations groups along the distribution range. This method is based on the null hypothesis that the genetic differentiation of outlier loci of one set of populations (with respect of genetic structure) is correlated with the background neutral genetic differentiation on the same set of populations. Thus, if the assumption is true, a correlation between Phi_(outlier)_ and Phi_(neutral)_) will be equal to 1, suggesting that the genetic differentiation of outlier loci in the set of populations can be explained by drift (rather than selection) at the local level (Gillespie & Oxford, 1998; Takahashi et al., 2014). Values significantly higher than 1 would indicate that the genetic differentiation of outlier loci is higher than the local background, suggesting local diversifying selection. Values significantly lower than 1 would indicate that the genetic differentiation of outlier loci is lower than the local background, suggesting local balancing selection. This method is analogous to that commonly used to evaluate the correlation between genetic and quantitative traits (McKay & Latta, 2002). We estimated the Phi_(outlier)_/Phi_(neutral)_ ratio at different spatial scales along the latitudinal environmental gradient: within Dson (Pops1–2), within a group combining the Dson and Dvov‐S populations (Pops1–3), within a group combining the Dvov‐S and Dvov‐N populations (Pops3–9), and within Dvov‐N (4–9). For each group, significant differentiation from a median ratio = 1 was estimated with a *t* test in the software PAST.

## 3 RESULTS

### 3.1 Species delimitation, genetic structure, and diversity

The species delimitation between *D. sonorense* (Pops 1–2) and *D. vovidesii* (Pops 3–9) was supported by all methods we used. STRUCTURE made a clear separation of the two species when considering the most optimum clustering, *K* = 2 (Figure 1 and Figure S2). This clustering clarifies that Pop3 is part of *D. vovidesii*, in concordance with our previous study analyzing the variation of the *trnL‐F* region of cpDNA (Gutiérrez‐Ortega, et al., 2014), thus resolving any doubt regarding its taxonomic status. This clustering also confirms an association between the species delimitation and the 28th parallel north. When considering *K* = 3 (the second most optimum clustering), *D. vovidesii* can be separated into two lineages that here we call Dvov‐S (Pop3) and Dvov‐N (Pops4–9). Similar lineage distinctions were also observed in the PCoA plots when considering the “all 871 loci,” the “809 neutral loci,” or the “62 outlier loci” datasets (Figure S3).

The TreeMix model that better fitted the data was the one containing no migration edges among populations (likelihood = 188.84). The topology of this tree (Figure 1c–d) showed that *D. sonorense* populations have experienced a lower degree of genetic drift than did all the *D. vovidesii* populations. Genetic drift has been particularly stronger in Dvov‐S, which might be associated with its low number of individuals (eight plants in 2016). The Z‐scores of the *f*3 statistic that evaluated whether any of the three lineages have experienced events of admixture from the two others were all positive: Z‐scores of Dson, Dvov‐S, and Dvov‐N were equal to 10.48, 13.26, and 5.98, respectively. Therefore, the neutral allelic frequencies found among populations can be explained solely by genetic drift, discarding the possibility of admixture after population/lineage divergence.

Using the neutral dataset, AMOVA showed that most of the neutral variation is found within populations (65%), whereas 17% of the variation occurs among populations and 18% between species (Table S6). Overall observed heterozygosity (*H_o_*) and expected heterozygosity (*H_e_*) were very similar between species (Table S7); *H_o_* and *H_e_* for *D. sonorense* were 0.238 and 0.254, whereas for *D. vovidesii* were 0.232 and 0.266. These values were also similar across populations, excepting Pop3, which showed the lowest level of heterozygosity (*H_o_* = 0.073 and *H_e_* = 0.044). In addition, we found that *H_o_* was higher than *H_e_* in all *D. vovidesii* populations, suggesting an excess of heterozygosity in this species. In contrast, *D. sonorense* populations did not show any excess of heterozygosity.

### 3.2 Demographic history

MIGRATE‐N estimated that the theta value (*Θ*) of *D. sonorense* (=0.0101) was lower than that of *D. vovidesii* (=0.0166) (Table S8). At the population level, *Θ* ranged from 0.0072 to 0.0223 (Table S9). When separating the dataset in the three lineages, *Θ* was estimated to be equal to 0.0099, 0.0075, and 0.0139 for Dson, Dvov‐S, and Dvov‐N, respectively (Table S10). In all cases, the number of migrants per generation (*Nm*) among populations, among lineages, and between the species was low, all with values <0.5 (Tables S8, S10, and S11), suggesting the absence of gene flow after speciation, after lineage split, or after population divergence.

**FIGURE 2:**
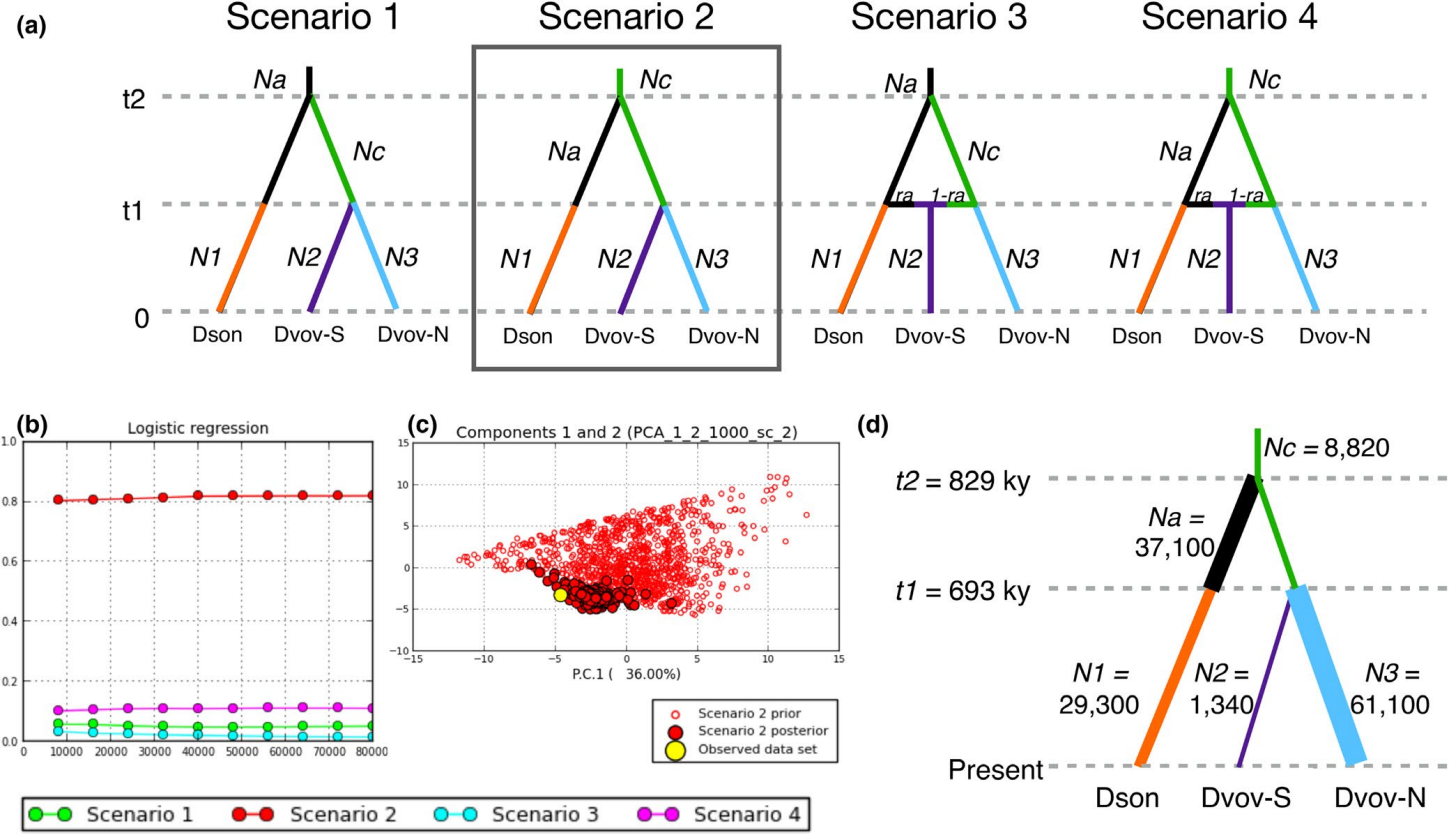
Four scenarios modeled in DIYABC (Cornuet et al., 2014). (a) Scenario 2 was selected as the best model according to the logistic regression test (b) implemented in DIYABC. (c) In Scenario 2, prior and posterior values were close to the observed dataset, supporting the certainty of the chosen scenario. (d) Graphical representation of Scenario 2 and parameter values estimated using the 1% of simulations. Time parameters *t1* and *t2* are given in thousand years before present, on the assumption of generation times of 100 years, as suggested by Vovides (1990). Abbreviations of parameters correspond to those shown in Figure S4 and Table S12

The approximate Bayesian computation method implemented in DIYABC was successful to compare four different scenarios that might explain the demographic history of the three lineages in the sister species pair *D. sonorense–D. vovidesii* (Figure 2a). Scenario 2 was found to be the most probable among the competitors (Figure 2b) and the posterior probabilities of its parameters were close to the observed data (Figure 2c), supporting the accuracy of the model. Scenario 2 suggests that a common ancestral lineage (*Nc* = 8,820; 95% confidence interval [CI] 4,600–16,900) had a demographic expansion that gave origin to an ancestral *D. sonorense* lineage (*Na* = 37,100; CI 7,890–64,600) *t2* generations ago (*t2* = 8,290; CI 5,080–12,700). Later, *t1* generations ago (*t1* = 6,930; CI 3,950–11,100), *Nc* had a population shrink that gave origin to the Dvov‐S lineage (*N2* = 1,340; CI 647–3,080) and a population expansion that gave origin to the Dvov‐N lineage (*N3* = 61,100; 95% CI) (Figure S4; Table S12). Assuming 100 years as generation time (Vovides, 1990), *t1* and *t2* would be equivalent to 693 and 829 ky, respectively.

### 3.3 Environmental variation between species' ranges

**FIGURE 3:**
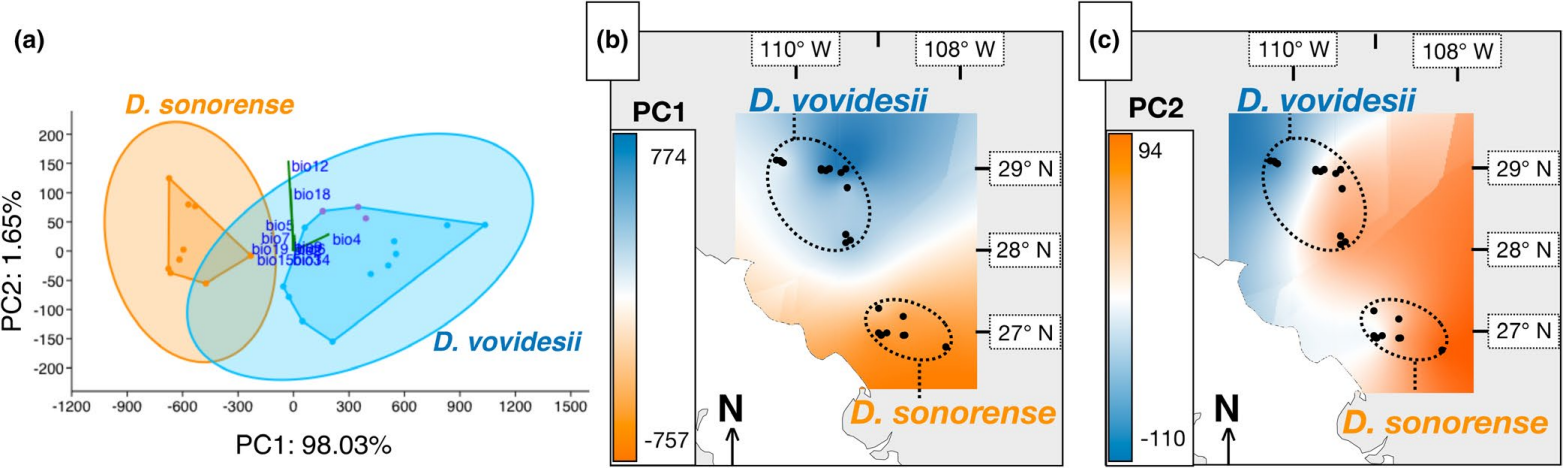
Environmental variation throughout the distribution range of *Dioon* *sonorense*–*D. vovidesii*. (a) Plot of the two main principal components (PCs) calculated from the variation of twelve noncorrelated environmental variables. Biplots indicate the relative contribution load of variables. The convex hulls and 95% ellipses of species are shown. Orange and blue dots indicate populations of *Dioon* *sonorense* (Dson) and *D. vovidesii* (Dvov‐N), respectively; purple dots indicate populations that occurred in the southernmost distribution limit of *D. vovidesii* (Dvov‐S). The percentage of explained variation of PCs is annotated at the axes labels. (b) Kriging interpolation of PC1 and (c) PC2 scores in the distribution range of the analyzed populations

The Welch's ANOVA and Kruskal–Wallis differentiation tests suggested that both species occur at nonsignificantly differentiated altitudes, ranging from 340 to 1,370 m above sea level. However, as expected due to the latitudinal variation, nine out of 19 bioclimatic variables are significantly differentiated between species (Table S13). *Dioon sonorense* inhabits less seasonal, warmer areas with higher isothermality (bio3, bio4, bio6, bio7). Also, the habitat of *D. sonorense* receives higher levels of precipitation than *D. vovidesii* (bio12, bio13, bio15, bio16, bio18). The PCA constructed from the variation of 12 noncorrelated bioclimatic variables estimated with 12 of the examined populations was able to summarize up to 99.68% of the total variation into only two PCs (Figure 3a). Biplots and loading scores (Table S15) indicate that PC1 and PC2 were mainly contributed by bio4 and bio12, respectively. The convex hulls of species do not overlap over the PC1, suggesting that PC1 is sufficient to separate the species. This is better noticed in the kriging interpolation maps: A clear environmental separation between the distribution ranges of the two species can be observed near the 28th parallel north in the kriging interpolation of the PC1 (Figure 3b), whereas PC2 separates the northernmost population occurring the in the most arid conditions of its range in the Sonoran Desert (Figure 3c).

**FIGURE 4:**
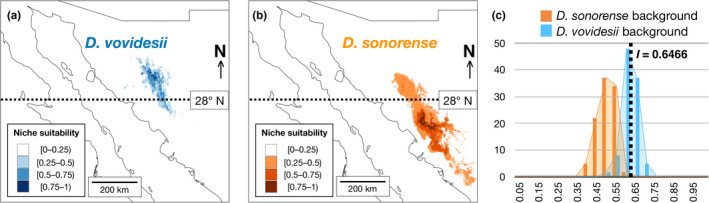
Ecological niche models for (a) *Dioon sonorense* and (b) *D. vovidesii*. Niche suitability of species was calculated in MaxEnt v3.3 (Phillips et al., 2006) using eight noncorrelated environment variables (Tables S13 and S14). (c) Empirical niche overlap, as estimated by the *I* index (Warren et al., 2008), is compared with the null distributions calculated in the background test implemented in ENMTOOLS (Warren et al., 2010)

The ENMs constructed for both species in MaxEnt showed high predictability and did not overfit the data, with AUC values of 0.983 ± 0.006 and 0.991 ± 0.002 for *D. sonorense* and *D. vovidesii*, respectively (Figure 4a–b). The niche suitability (>.25) of *D. vovidesii* was mostly restricted to the north of the 28th parallel north, but the niche suitability (>.25) of *D. sonorense* extends toward the distribution range of *D. vovidesii,* to the north of the 28th parallel north. The differences on the extensions of niche suitability are indicated by the niche breadth estimations using the B1 (inverse concentration) metric, which was lower in *D. vovidesii* (0.0913) than it was in *D. sonorense* (0.2009).

For both species, bio4 (temperature seasonality), bio5 (max temperature of the warmest month), and bio19 (precipitation of coldest quarter) were the variables that contributed the most for the ENM construction (Table S14), suggesting these variables as common limiting factors in the distribution of *Dioon* in Sonora, Mexico. In addition, bio2 (mean diurnal range temperature) resulted to be informative for the predictability of *D. sonorense* but not for *D. vovidesii*, whereas bio6 (min temperature of the coldest month) was informative for *D. vovidesii*, but not for *D. sonorense*. The empirical overlap between ENM of the two species was estimated as *I* = 0.6466. In the background test, this empirical *I* index value was equivalent to the null distribution constructed by comparing the ENM of *D. sonorense* with ENMs constructed from random points sampled from the *D. vovidesii* background (*p* > .05). However, the *I* index was significantly higher than the null distribution constructed by comparing the overlap between the ENM of *D. vovidesii* and 100 ENM replicates constructed of random points sampled from the background of *D. sonorense* (*p* = 0) (Figure 4c). This latter result indicates that despite speciation, both species retain the same ancestral niche.

### 3.4 Influence of the latitudinal environmental gradient on the genetic variation

**FIGURE 5:**
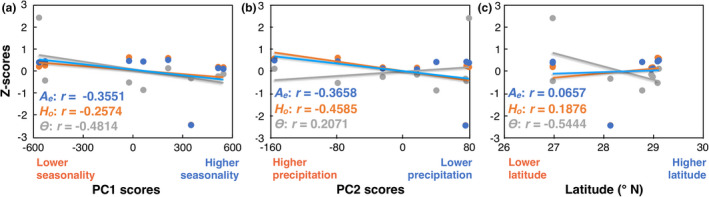
Linear correlations between genetic diversity parameters and environmental variables. Correlations were estimated with PC1 scores (a), PC2 scores (b), and latitude (c). The number of effective alleles (*A_e_*) and observed heterozygosity (*H_o_*) was estimated in GenAlEx; *Θ* values correspond to those obtained in MIGRATE‐N analysis (Table S9)

We found nonsignificant correlations between genetic diversity parameters (*A_e_*, *H_o_*, and *Θ*) and latitude, or PC1 and PC2 scores (Figure 5). However, there are negative correlations between the three parameters of genetic diversity and PC1. PC1 is almost completely contributed by bio4 (Figure 3b; Table S15); thus, these results suggest that less seasonal populations tend to have higher levels of genetic diversity. The correlations between the genetic diversity parameters with PC2 or latitudinal differentiation were inconsistently negative or positive.

For the “All loci” and “Neutral loci” matrices of genetic differentiation, the best linear regression model was that including the effect of the latitudinal differentiation plus the geographic distances and their interaction as the explanatory variables (Table 2). If we added any environmental variables (i.e., environmental dissimilarity, bio4, or PC1) into the models, the fitness of the data was decreased (Table S16). We interpret these results that the neutral divergence among populations is mainly contributed by geographic isolation rather than the environmental variation. However, the "Outlier loci” matrix showed a different result, since the best model also includes the effect of bio4 (temperature seasonality) as an explanatory variable (Table 2). These results suggest that whereas geographic component associated with latitude influences the neutral divergence between the species, the latitude‐associated seasonality influences disparate local selective pressures on the two species.

**TABLE 2:** The best linear regression models explaining the genetic differentiation among populations

Dataset	Best model	Intercept	*SE*	*t*‐value	Pr(>|*t*|)	AICc
Neutral loci	Lat*Geo	1.0504	0.1599	6.570	8.54E−07	46.42471
All loci	Lat*Geo	1.0228	0.1622	6.307	1.61E−06	47.2178
Outlier loci	Lat*Geo + bio4	0.8014	0.1636	4.898	6.01E−05	49.79075

Note

The effect of latitudinal differentiation, the geographic distances, and their interaction (Lat*Geo models) best fitted the model to explain the “Neutral" and “All loci” genetic differentiation among populations. Besides, the best model of the "Outlier loci” matrix additionally includes the effect of temperature seasonality (bio4) as an explanatory variable. Detailed results from linear regression models can be found in Table S16. AICc = corrected Akaike information criterion.

**FIGURE 6:**
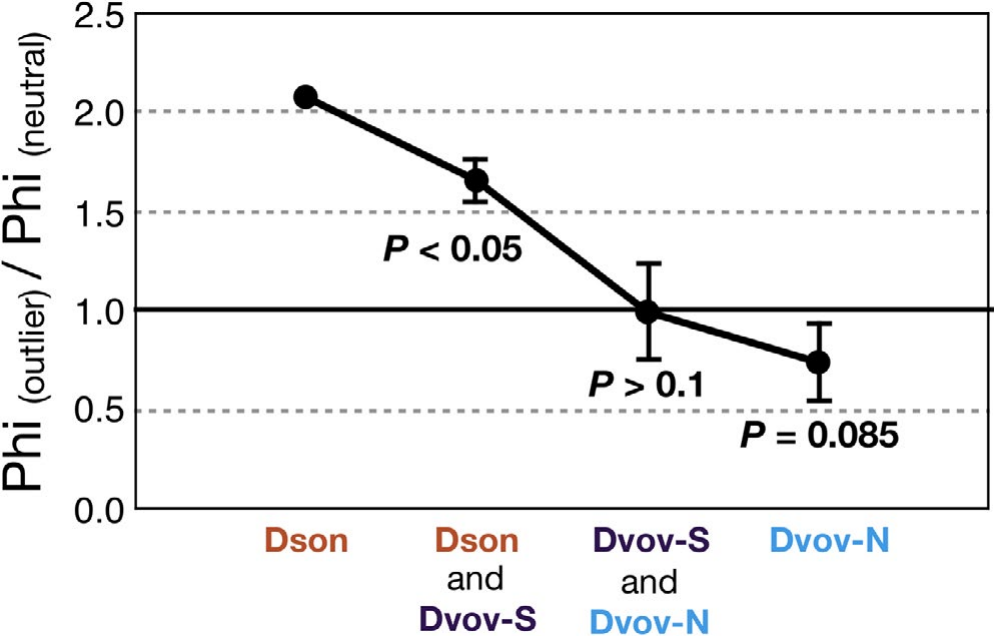
Variation of the Phi_(outlier)_/Phi_(neutral)_ ratio and standard deviations across population groups along the latitudinal gradient, from the south (left) to north (right). Dson = Pops1–2; Dson and Dvov‐S = Pops1–3; Dvov‐S and Dvov‐N = Pops3–9; and Dvov‐N = Pops4–9. The hypothesis that non‐neutral differentiation can be explained by simple drift cannot be discarded if the ratios are equal to 1. Diversifying or balancing selection can explain the cases when the ratio is higher or lower than 1, respectively. Significant departure from mean ratio = 1 was tested with the *t* test; *p*‐values are specified. The *t* test could not be performed for the population group Dson (Pops1–2) due to low sample size (one pairwise comparison)

The Phi_(outlier)_/Phi_(neutral)_ ratio was uneven among neighboring population groups throughout latitude (Figure 6). At lower latitudes, within both Dson and a group formed by Dson and Dvov‐S, the ratios (and standard deviations) are higher than 1, suggesting that the non‐neutral population differentiation among these populations might not be explained by simple drift (i.e., diversifying selection might be relatively stronger at lower latitudes). When combining Dvov‐S and Dvov‐N populations, the ratio is not significantly different to a mean = 1. When considering only the Dvov‐N populations, the ratio is marginally significant (*p* = .085), suggesting a stronger effect of balancing selection at higher latitudes.

## 4 DISCUSSION

### 4.1 Historical process of species divergence

This study suggests that the combination of geographic isolation and the effect of the latitudinal environmental gradient (temperature seasonality, in particular) in northwestern Mexico promoted the divergence between *D. sonorense* and *D. vovidesii*. This sister species pair occurs at the northern margin of the genus *Dioon*, across the Nearctic–Neotropical transition zone in Sonora, Mexico. Despite the absence of a clear geographic barrier, genetic and environmental analyses identified that the separation between species coincides with the 28th parallel north (Figure 1a), in concordance with previous observations regarding the limits of the two biogeographic zones (Martínez‐Yrízar et al., 2000).

The best‐supported ABC model (Scenario 2) suggests that all Sonoran lineages of *Dioon* share a common ancestor (Figure 2a) that dispersed along the gradient prior to its first split, ~829 ky ago, and a second split a short time later, ~693 ky ago. The dispersal of the ancestral population is likely to have occurred through a stepping‐stone mode of short distance dispersal (Gutiérrez‐Ortega, Salinas‐Rodríguez, et al., 2018) throughout a continuous Neotropical habitat that reached central Sonora before the mid‐Pleistocene climate fluctuations. Currently, the northernmost populations of *D. vovidesii* occur in Sierra de Mazatán near the 29th parallel north, an isolated mountain embedded by xeric vegetation communities belonging to the Sonoran Desert (Piña‐Páez et al., 2013; Sánchez‐Escalante et al., 2017). This particular occurrence suggests the existence of an ancestral corridor that allowed the dispersal of *Dioon* before the aridification of this region. The Sonoran Desert originated around 2.5 Ma but expanded rapidly in the last 1 Ma (Smith & Farrell, 2005; Van Devender et al.,^1987^, ^1994^), reaching areas where now *D. vovidesii* occurs, in agreement with times of lineage divergence in this sister pair (Figure 2). As suggested in a previous phylogenetic and epidermal anatomy study (Gutiérrez‐Ortega, Yamamoto et al., 2018), the expansion of arid zones in Sonora might be responsible for the habitat fragmentation of the Neotropical forests and the hampered gene flow among *Dioon* populations.

### 4.2 Effect of the latitudinal environmental gradient on the genetic variation

The distinction between *D. sonorense* and *D. vovidesii* was associated with the latitudinal environmental gradient in multiple abiotic aspects. First, as predicted by Janzen (1967), our results on climatic data show that the northern species, *D. vovidesii*, occurs in more seasonal climates, with warmer, drier habitats, and colder winters than does the southern species *D. sonorense* (Table S13). Indeed, PC1 is almost completely loaded by bio4 (temperature seasonality) and it can solely make a distinction between the two species (Figure 1a and Figure 3b). Second, the correlation tests between genetic diversity parameters and the PC1 were all negative (nonsignificant though), suggesting that less seasonal populations (the more southern populations) tend to have higher levels of diversity (Figure 5; Table S7 and Table S9). Third, the linear models demonstrated that the neutral genetic differentiation among populations can be explained by the effect of geographic isolation and its interaction with the latitudinal differentiation, whereas the genetic differentiation of loci putatively under selection is reinforced by the addition of the temperature seasonality (bio4) (Table 2 and Table S16).

Despite the clear environmental differentiation along the latitudinal environmental gradient, the background test of niche similarity using ENMs suggested niche conservatism between species (Figure 4c). Niche conservatism has also been found in another case of speciation in *Dioon* (Gutiérrez‐Ortega, Salinas‐Rodríguez, et al., 2020), supporting the idea that niche conservatism is a common factor involved in cycad speciation. In this case, niche conservatism suggests that *D. sonorense* and *D. vovidesii*, although living in different environmental conditions, share common ecological factors limiting their distribution. This can be observed in the jackknife test obtained in MaxEnt, which shows that bio4, bio5, and bio19 have the greatest contribution for the prediction on the distribution of both species (Table S14). Also, it might indicate that *D. vovidesii* populations are tolerating, rather than utilizing the novel environmental conditions presented at high latitudes, as discussed below.

Populations of tropical species at higher latitudes are expected to show lower levels of genetic diversity because they might have been subjected to founder effects or more drastic fluctuations of population size than do the southern populations (Cortázar‐Chinarro et al., 2017); this might be true for this case. Although values are nonsignificant, correlation tests suggest that populations at higher latitudes and more seasonal environments tend to have lower levels of genetic diversity (Figure 5). Also, the maximum‐likelihood tree produced in TreeMix (Figure 1c) suggests that *D. vovidesii* populations might have experienced stronger genetic drift than did the *D. sonorense* populations. This can be explained by the contrasting demographic trends between species: While the Dson lineage had a slight decrement of effective population size at *t1*, Dvov‐N had a drastic increment (Figure 2).

The excess of heterozygosity in all *D. vovidesii* populations might indicate that balancing selection might be involved in the genetic variation of this species (Cornuet & Luikart, 1996). The estimations of the Phi_(outlier)_/Phi_(neutral)_ ratio in the Dvov‐N lineage were <1, suggesting that at the local level, non‐neutral loci are less differentiated among populations than the neutral loci, although this value was marginally significantly lower than 1 (*p* = .085; Figure 6). In contrast, we found that this selection regime of balancing selection is not occurring in populations at lower latitudes. In *D. sonorense*, the ratio was >2, indicating that diversifying selection is more likely to be acting within this southern species. Indeed, we found a clinal decrement on the Phi_(outlier)_/Phi_(neutral)_ among populations groups along the gradient (Figure 6). This result agrees with observations by Takahashi et al. (2014), who suggested that selective pressures at different local scales can vary with latitude. Although this is a speculation, these clues of balancing selection in the northern populations of *D. vovidesii* might suggest that high‐latitude populations have maintained advantageous genetic variation to tolerate unfavorable environmental conditions associated with strong seasonality (Delph & Kelly, 2014). Future studies should analyze whether the resilience of cycad populations at apparently harsh environmental conditions is due to the effect of balancing selection.

### 4.3 Parapatric or allopatric speciation?

A final question is whether the case of speciation we are documenting can be explained by the parapatric or the allopatric speciation models. Certainly, the current parapatric geographic distribution of the species does not imply parapatric speciation (Endler, 1982); however, there are pieces of evidence that allow us to speculate that parapatric speciation has been at least plausible and that it might be useful to consider this study system in future investigations.

First, parapatric speciation is often thought to be accompanied by local adaptation (Coyne & Orr, 2004; Endler, 1982). This seems to be the case for *D. sonorense–D. vovidesii*. Our linear models suggest that environmental variation influenced the genetic differentiation among populations when considering only outlier loci (Table 2), and we also detected clues of contrasting selection regimes on population groups (Figure 6). Even if selection is not considered, parapatric speciation can also occur solely by demographic factors, especially for neighboring lineages with small population sizes and reduced dispersal capabilities (Gavrilets et al., 2000) as in the case of *Dioon* due to their large and heavy seeds (Vovides, 1990). However, we cannot provide evidence that the contrasting natural selection regimes between species that we detected in this study also occurred during the times of divergence, ~829 ky ago, and we cannot preclude the possibility that the contrasting natural selection regimes we observe now are just by‐products of the long‐term isolation in different environments after completed divergence. Additionally, in the parapatric speciation model, it is expected that divergence between lineages occurred due to primary parapatric divergence, where an intermediate population, such as Pop3, maintained gene flow during the speciation (Gavrilets et al., 2000). Our results preclude the possibility of secondary contact (Figures 1, 2, 3; Tables S8, S10–S12), suggesting that genetic drift and the influence of local environmental variation has caused the divergence. However, our dataset is insufficient to distinguish whether divergence between the two species occurred in the presence of gene flow via the intermediate populations.

In light of the presented evidence, we can suggest that the allopatric speciation model is the simplest explanation for the origin of *D. sonorense–D. vovidesii*. Our results indicate that speciation along the latitudinal environmental gradient was triggered due to the combination of demographic factors, low dispersal capabilities to maintain interpopulation connectivities along the gradient, and increasing population fragmentation, isolation, and disparate selection due to the expansion of the drier and seasonal climates at the northern region of northwestern Mexico.

## 5 CONCLUSION

This study represents an empirical example of how speciation can be achieved through the effect of the latitudinal variation among wild populations. As theory predicts, latitude provided a geographic scene favorable for lineage divergence, in which geographic isolation facilitates neutral divergence, while the effect of seasonality has an additive divergent effect because it promotes contrasting selection regimes. The latitudinal gradient provided a sharp variation in climate seasonality that influenced disparate demographic histories at either side of the 28th parallel north. Such influence of latitude‐associated environmental variation on speciation was predicted by Janzen (1967) and seconded by Kozak and Wiens (2007); thus, we agree that climatic zonation in latitudinal gradients has played important roles in the speciation of tropical groups. Finally, the genus *Dioon* is well recognized for being a well representor of the Neotropical biota in the Mexican transition zone. Thus, since the two species we studied occur at the limits between the Nearctic and the Neotropical biogeographic zones, our study also adds implications on how the margins of distribution of biological groups (a tropical plant genus in this case) are shaped.

## CONFLICT OF INTEREST

None declared.

## AUTHOR CONTRIBUTIONS

**José Said Gutiérrez‐Ortega:** Conceptualization (lead); data curation (lead); formal analysis (lead); funding acquisition (lead); investigation (lead); methodology (lead); project administration (lead); resources (lead); software (lead); visualization (lead); writing–original draft (lead); writing–review and editing (lead). **Francisco Molina‐Freaner:** Resources (supporting); validation (equal); writing–review and editing (equal). **José F. Martínez:** Methodology (supporting); resources (supporting). **Miguel Angel Pérez‐Farrera:** Resources (supporting); validation (supporting); writing–review and editing (supporting). **Andrew Vovides:** Validation (supporting); writing–review and editing (supporting). **Antonio Hernández‐López:** Validation (supporting); writing–review and editing (supporting). **Ayumi Tezuka:** Data curation (supporting); methodology (supporting). **Atsushi J. Nagano:** Data curation (supporting); methodology (supporting); resources (supporting). **Yasuyuki Watano:** Supervision (supporting); validation (supporting); writing–review and editing (supporting). **Yuma Takahashi:** Validation (supporting); writing–review and editing (supporting). **Masashi Murakami:** Methodology (supporting); project administration (supporting); supervision (supporting); validation (supporting); writing–review and editing (supporting). **Tadashi Kajita:** Conceptualization (supporting); project administration (supporting); supervision (lead); validation (supporting); visualization (supporting); writing–review and editing (supporting).

## Supporting information

Supplementary Material

## Data Availability

Illumina raw reads produced in ddRAD‐Seq, the SNP datasets in STRUCTURE format, and environmental data of localities of the analyzed populations are publicly available on the Figshare depository https://doi.org/10.6084/m9.figshare.14227715.v1 (Gutiérrez‐Ortega, Molina‐Freaner, et al., 2020).
