# Supplementary material for: Speciation along a latitudinal gradient: The origin of the Neotropical cycad sister pair Dioon sonorense–D. vovidesii (Zamiaceae)
Source: Ecol Evol. 2021 May 1;11(11):6962–76. doi: 10.1002/ece3.7545 (PMC8207156; doi:10.1002/ece3.7545)
Supplement: Supplementary file 1 — Supplementary Material [file ECE3-11-6962-s001.docx]

Table S1. List of samples used in this study. Population (Pop), sample file names, and sample labels (ID) are listed. The number of reads and total bases obtained from RAD-seq, GC and AT content percentage, and quality evaluated from Q20 and Q30% are also indicated.

| Pop | ID | Read Count | Total Bases | GC (%) | AT (%) | Q20 (%) | Q30 (%) |
| --- | --- | --- | --- | --- | --- | --- | --- |
| 1 | Pop1_1 | 1,229,882 | 62,723,982 | 36.39 | 63.61 | 96.84 | 94.79 |
| 1 | Pop1_2 | 2,005,010 | 102,255,510 | 36.12 | 63.88 | 96.91 | 94.82 |
| 1 | Pop1_3 | 2,103,246 | 107,265,546 | 36.15 | 63.85 | 97.17 | 95.15 |
| 1 | Pop1_4 | 2,278,987 | 116,228,337 | 36.14 | 63.86 | 96.88 | 94.83 |
| 1 | Pop1_5 | 2,385,263 | 121,648,413 | 36.54 | 63.46 | 96.87 | 94.81 |
| 1 | Pop1_6 | 2,410,420 | 122,931,420 | 35.75 | 64.25 | 96.85 | 94.73 |
| 1 | Pop1_7 | 2,494,439 | 127,216,389 | 36.31 | 63.69 | 96.92 | 94.86 |
| 1 | Pop1_8 | 2,558,600 | 130,488,600 | 36.12 | 63.88 | 96.93 | 94.89 |
| 1 | Pop1_9 | 2,646,888 | 134,991,288 | 35.97 | 64.03 | 97.46 | 95.41 |
| 1 | Pop1_10 | 2,872,906 | 146,518,206 | 36.08 | 63.92 | 96.94 | 94.89 |
| 1 | Pop1_11 | 2,904,894 | 148,149,594 | 36.0 | 64.0 | 97.1 | 95.06 |
| 1 | Pop1_12 | 3,249,139 | 165,706,089 | 36.0 | 64.0 | 96.88 | 94.79 |
| 1 | Pop1_13 | 3,484,309 | 177,699,759 | 35.85 | 64.15 | 96.9 | 94.8 |
| 1 | Pop1_14 | 3,736,339 | 190,553,289 | 36.13 | 63.87 | 97.01 | 95.01 |
| 1 | Pop1_15 | 3,955,315 | 201,721,065 | 36.45 | 63.55 | 97.03 | 94.99 |
| 1 | Pop1_16 | 4,017,153 | 204,874,803 | 36.46 | 63.54 | 96.99 | 94.92 |
| 2 | Pop2_1 | 1,613,265 | 82,276,515 | 36.4 | 63.6 | 96.94 | 94.89 |
| 2 | Pop2_2 | 1,895,535 | 96,672,285 | 36.16 | 63.84 | 96.87 | 94.82 |
| 2 | Pop2_3 | 1,923,147 | 98,080,497 | 36.34 | 63.66 | 96.95 | 94.92 |
| 2 | Pop2_4 | 1,945,419 | 99,216,369 | 35.85 | 64.15 | 97.0 | 94.84 |
| 2 | Pop2_5 | 2,079,953 | 106,077,603 | 35.91 | 64.09 | 97.05 | 95.0 |
| 2 | Pop2_6 | 2,320,012 | 118,320,612 | 36.03 | 63.97 | 96.95 | 94.89 |
| 2 | Pop2_7 | 2,466,032 | 125,767,632 | 36.32 | 63.68 | 96.92 | 94.83 |
| 2 | Pop2_8 | 2,474,204 | 126,184,404 | 35.59 | 64.41 | 96.93 | 94.84 |
| 2 | Pop2_9 | 2,531,916 | 129,127,716 | 35.92 | 64.08 | 97.02 | 94.98 |
| 2 | Pop2_10 | 2,738,241 | 139,650,291 | 36.27 | 63.73 | 96.89 | 94.81 |
| 2 | Pop2_11 | 2,751,031 | 140,302,581 | 36.38 | 63.62 | 96.87 | 94.77 |
| 2 | Pop2_12 | 2,778,488 | 141,702,888 | 35.69 | 64.31 | 96.99 | 94.9 |
| 2 | Pop2_13 | 2,873,170 | 146,531,670 | 35.94 | 64.06 | 96.99 | 94.92 |
| 2 | Pop2_14 | 2,955,300 | 150,720,300 | 35.99 | 64.01 | 96.99 | 94.93 |
| 2 | Pop2_15 | 3,375,569 | 172,154,019 | 36.1 | 63.9 | 96.94 | 94.9 |
| 2 | Pop2_16 | 3,591,147 | 183,148,497 | 36.41 | 63.59 | 96.94 | 94.89 |
| 3 | Pop3_1 | 2,018,291 | 102,932,841 | 36.27 | 63.73 | 96.98 | 94.93 |
| 3 | Pop3_2 | 2,254,122 | 114,960,222 | 36.41 | 63.59 | 96.88 | 94.77 |
| 3 | Pop3_3 | 3,389,866 | 172,883,166 | 36.13 | 63.87 | 96.88 | 94.8 |
| 3 | Pop3_4 | 3,567,815 | 181,958,565 | 36.45 | 63.55 | 96.89 | 94.81 |
| 3 | Pop3_5 | 3,981,142 | 203,038,242 | 36.73 | 63.27 | 96.96 | 94.91 |
| 4 | Pop4_1 | 1,372,717 | 70,008,567 | 36.25 | 63.75 | 96.89 | 94.85 |
| 4 | Pop4_2 | 1,481,113 | 75,536,763 | 36.04 | 63.96 | 96.86 | 94.82 |
| 4 | Pop4_3 | 1,642,024 | 83,743,224 | 36.01 | 63.99 | 96.91 | 94.84 |
| 4 | Pop4_4 | 1,884,843 | 96,126,993 | 36.23 | 63.77 | 96.89 | 94.85 |
| 4 | Pop4_5 | 2,192,373 | 111,811,023 | 35.36 | 64.64 | 97.09 | 94.99 |
| 4 | Pop4_6 | 2,282,330 | 116,398,830 | 36.07 | 63.93 | 97.07 | 95.03 |
| 4 | Pop4_7 | 2,385,893 | 121,680,543 | 35.98 | 64.02 | 97.05 | 94.97 |
| 4 | Pop4_8 | 2,391,443 | 121,963,593 | 36.14 | 63.86 | 97.02 | 94.94 |
| 4 | Pop4_9 | 2,460,282 | 125,474,382 | 36.1 | 63.9 | 96.83 | 94.72 |
| 4 | Pop4_10 | 2,499,771 | 127,488,321 | 36.35 | 63.65 | 96.73 | 94.49 |
| 4 | Pop4_11 | 2,590,421 | 132,111,471 | 36.18 | 63.82 | 96.9 | 94.83 |
| 4 | Pop4_12 | 2,762,472 | 140,886,072 | 35.94 | 64.06 | 96.93 | 94.86 |
| 4 | Pop4_13 | 3,173,198 | 161,833,098 | 36.43 | 63.57 | 96.9 | 94.84 |
| 4 | Pop4_14 | 3,179,250 | 162,141,750 | 36.23 | 63.77 | 96.88 | 94.82 |
| 4 | Pop4_15 | 3,399,231 | 173,360,781 | 36.32 | 63.68 | 96.98 | 94.95 |
| 4 | Pop4_16 | 3,678,846 | 187,621,146 | 36.12 | 63.88 | 96.95 | 94.92 |
| 5 | Pop5_1 | 1,014,941 | 51,761,991 | 36.4 | 63.6 | 96.81 | 94.69 |
| 5 | Pop5_2 | 1,775,726 | 90,562,026 | 36.48 | 63.52 | 96.9 | 94.84 |
| 5 | Pop5_3 | 1,808,113 | 92,213,763 | 36.29 | 63.71 | 97.0 | 94.99 |
| 5 | Pop5_4 | 2,261,442 | 115,333,542 | 36.27 | 63.73 | 96.86 | 94.79 |
| 5 | Pop5_5 | 2,273,399 | 115,943,349 | 36.26 | 63.74 | 96.91 | 94.85 |
| 6 | Pop6_1 | 3,302,416 | 168,423,216 | 35.62 | 64.38 | 96.92 | 94.85 |
| 6 | Pop6_2 | 4,647,193 | 237,006,843 | 36.45 | 63.55 | 96.97 | 94.94 |
| 7 | Pop7_1 | 1,662,191 | 84,771,741 | 35.98 | 64.02 | 96.82 | 94.62 |
| 7 | Pop7_2 | 1,974,308 | 100,689,708 | 36.03 | 63.97 | 96.69 | 94.48 |
| 7 | Pop7_3 | 1,996,853 | 101,839,503 | 36.23 | 63.77 | 96.95 | 94.92 |
| 7 | Pop7_4 | 2,237,672 | 114,121,272 | 36.12 | 63.88 | 96.85 | 94.73 |
| 7 | Pop7_5 | 2,668,925 | 136,115,175 | 35.88 | 64.12 | 96.86 | 94.72 |
| 7 | Pop7_6 | 2,711,887 | 138,306,237 | 36.01 | 63.99 | 96.88 | 94.76 |
| 7 | Pop7_7 | 2,752,066 | 140,355,366 | 36.15 | 63.85 | 96.89 | 94.8 |
| 7 | Pop7_8 | 3,091,352 | 157,658,952 | 35.77 | 64.23 | 96.96 | 94.92 |
| 8 | Pop8_1 | 1,148,757 | 58,586,607 | 36.27 | 63.73 | 97.03 | 95.02 |
| 8 | Pop8_2 | 2,004,749 | 102,242,199 | 36.19 | 63.81 | 96.86 | 94.76 |
| 8 | Pop8_3 | 2,077,586 | 105,956,886 | 36.39 | 63.61 | 96.89 | 94.81 |
| 8 | Pop8_4 | 2,506,851 | 127,849,401 | 36.29 | 63.71 | 96.96 | 94.88 |
| 8 | Pop8_5 | 2,689,072 | 137,142,672 | 35.92 | 64.08 | 96.99 | 94.95 |
| 8 | Pop8_6 | 2,971,376 | 151,540,176 | 36.22 | 63.78 | 96.93 | 94.84 |
| 8 | Pop8_7 | 3,018,237 | 153,930,087 | 36.23 | 63.77 | 97.05 | 94.9 |
| 8 | Pop8_8 | 3,077,325 | 156,943,575 | 35.95 | 64.05 | 96.82 | 94.68 |
| 8 | Pop8_9 | 3,134,136 | 159,840,936 | 36.17 | 63.83 | 96.98 | 94.98 |
| 8 | Pop8_10 | 3,230,052 | 164,732,652 | 36.27 | 63.73 | 96.83 | 94.72 |
| 9 | Pop9_1 | 1,611,734 | 82,198,434 | 36.01 | 63.99 | 96.82 | 94.74 |
| 9 | Pop9_2 | 2,170,637 | 110,702,487 | 36.25 | 63.75 | 96.85 | 94.71 |
| 9 | Pop9_3 | 2,287,403 | 116,657,553 | 36.06 | 63.94 | 97.11 | 95.09 |
| 9 | Pop9_4 | 2,566,808 | 130,907,208 | 35.97 | 64.03 | 96.77 | 94.71 |
| 9 | Pop9_5 | 2,600,591 | 132,630,141 | 36.14 | 63.86 | 96.97 | 94.9 |
| 9 | Pop9_6 | 2,675,086 | 136,429,386 | 36.03 | 63.97 | 97.12 | 95.1 |
| 9 | Pop9_7 | 2,732,685 | 139,366,935 | 36.22 | 63.78 | 96.95 | 94.89 |
| 9 | Pop9_8 | 2,924,176 | 149,132,976 | 35.93 | 64.07 | 96.99 | 94.87 |
| 9 | Pop9_9 | 2,956,050 | 150,758,550 | 35.99 | 64.01 | 96.96 | 94.92 |
| 9 | Pop9_10 | 3,528,568 | 179,956,968 | 36.09 | 63.91 | 96.91 | 94.87 |
| 9 | Pop9_11 | 4,118,383 | 210,037,533 | 36.42 | 63.58 | 96.84 | 94.69 |

Table S2. Set of physiographic provinces considered in the ecological niche modeling methods. Provinces, as defined by Cervantes-Zamora et al. (1990), correspond to areas where *D. vovidesii*, *D. sonorense*, and the closely related species *D. tomasellii* occur.

| **Province** | **Species** |
| --- | --- |
| Provincia Sierras y Llanuras Sonorenses | *D. vovidesii* |
| Provincia Sierras y Valle del Norte | *D. vovidesii* |
| Provincia Sierras y Cañada del Norte | *D. vovidesii* |
| Provincia Pie de la Sierra | *D. vovidesii* and *D. sonorense* |
| Provincia Gran Meseta y Cañones Chihuahuenses | *D. sonorense* |
| Provincia Gran Meseta y Cañones Duranguenses | *D. tomasellii* |
| Provincia Mesetas y Cañadas del Sur | *D. tomasellii* |
| Provincia Sierras y Valles Zacatecanos | *D. tomasellii* |
| Provincia Sierras Neovolcánicas Nayaritas | *D. tomasellii* |
| Provincia Guadalajara | *D. tomasellii* |
| Provincia Sierra de Jalisco | *D. tomasellii* |
| Provincia Sierras de la Costa de Jalisco y Colima | *D. tomasellii* |

Table S3. Pearson’s correlations *r* values among variables in the set of biogeographic provinces used for ENMs. Values r > 0.9 or <-0.9 are highlighted. Variables in red cells were removed in ENM constructions.

|  | bio2 | bio3 | bio4 | bio5 | bio6 | bio7 | bio8 | bio9 | bio10 | bio11 | bio12 | bio13 | bio14 | bio15 | bio16 | bio17 | bio18 | bio19 |
| --- | --- | --- | --- | --- | --- | --- | --- | --- | --- | --- | --- | --- | --- | --- | --- | --- | --- | --- |
| bio1 | -0.086 | 0.124 | 0.053 | 0.822 | 0.873 | -0.039 | 0.905 | 0.956 | 0.909 | 0.923 | -0.008 | 0.088 | -0.561 | 0.518 | 0.090 | -0.542 | 0.040 | -0.289 |
| bio2 |  | -0.065 | 0.592 | 0.406 | -0.507 | 0.871 | 0.148 | -0.054 | 0.165 | -0.314 | -0.396 | -0.356 | 0.171 | -0.016 | -0.408 | 0.074 | -0.302 | -0.050 |
| bio3 |  |  | -0.714 | -0.185 | 0.322 | -0.483 | -0.195 | -0.016 | -0.188 | 0.391 | 0.650 | 0.627 | 0.149 | 0.526 | 0.662 | 0.118 | 0.544 | -0.033 |
| bio4 |  |  |  | 0.558 | -0.384 | 0.899 | 0.465 | 0.193 | 0.463 | -0.335 | -0.722 | -0.681 | -0.117 | -0.301 | -0.729 | -0.117 | -0.550 | 0.023 |
| bio5 |  |  |  |  | 0.450 | 0.532 | 0.949 | 0.847 | 0.960 | 0.559 | -0.362 | -0.257 | -0.462 | 0.305 | -0.291 | -0.460 | -0.257 | -0.239 |
| bio6 |  |  |  |  |  | -0.516 | 0.619 | 0.793 | 0.617 | 0.974 | 0.296 | 0.356 | -0.501 | 0.526 | 0.385 | -0.458 | 0.266 | -0.241 |
| bio7 |  |  |  |  |  |  | 0.324 | 0.061 | 0.336 | -0.388 | -0.627 | -0.584 | 0.032 | -0.206 | -0.644 | -0.007 | -0.498 | -0.001 |
| bio8 |  |  |  |  |  |  |  | 0.930 | 0.998 | 0.677 | -0.318 | -0.218 | -0.560 | 0.320 | -0.233 | -0.549 | -0.196 | -0.263 |
| bio9 |  |  |  |  |  |  |  |  | 0.930 | 0.829 | -0.129 | -0.049 | -0.567 | 0.353 | -0.047 | -0.556 | -0.090 | -0.246 |
| bio10 |  |  |  |  |  |  |  |  |  | 0.680 | -0.314 | -0.212 | -0.556 | 0.329 | -0.229 | -0.541 | -0.199 | -0.261 |
| bio11 |  |  |  |  |  |  |  |  |  |  | 0.269 | 0.345 | -0.488 | 0.602 | 0.366 | -0.472 | 0.252 | -0.288 |
| bio12 |  |  |  |  |  |  |  |  |  |  |  | 0.977 | 0.431 | 0.351 | 0.984 | 0.515 | 0.897 | 0.441 |
| bio13 |  |  |  |  |  |  |  |  |  |  |  |  | 0.358 | 0.477 | 0.988 | 0.443 | 0.914 | 0.370 |
| bio14 |  |  |  |  |  |  |  |  |  |  |  |  |  | -0.235 | 0.319 | 0.920 | 0.385 | 0.683 |
| bio15 |  |  |  |  |  |  |  |  |  |  |  |  |  |  | 0.464 | -0.233 | 0.411 | -0.271 |
| bio16 |  |  |  |  |  |  |  |  |  |  |  |  |  |  |  | 0.393 | 0.911 | 0.322 |
| bio17 |  |  |  |  |  |  |  |  |  |  |  |  |  |  |  |  | 0.461 | 0.824 |
| bio18 |  |  |  |  |  |  |  |  |  |  |  |  |  |  |  |  |  | 0.384 |

Table S4. Pairwise population differentiation of parameters used for correlation tests. Values are annotated below diagonal and *P* values are above the diagonal, when applicable.

| A) Phi-values (809 neutral loci) | | | | | | | | |
| --- | --- | --- | --- | --- | --- | --- | --- | --- |
|  | Pop 1 | Pop 2 | Pop 3 | Pop 4 | Pop 5+6 | Pop 7 | Pop 8 | Pop 9 |
| Pop 1 |  | 0.001 | 0.001 | 0.001 | 0.001 | 0.001 | 0.001 | 0.001 |
| Pop 2 | 0.118 |  | 0.001 | 0.001 | 0.001 | 0.001 | 0.001 | 0.001 |
| Pop 3 | 0.494 | 0.529 |  | 0.001 | 0.001 | 0.001 | 0.002 | 0.001 |
| Pop 4 | 0.309 | 0.347 | 0.486 |  | 0.001 | 0.001 | 0.001 | 0.001 |
| Pop 5+6 | 0.325 | 0.369 | 0.575 | 0.126 |  | 0.001 | 0.001 | 0.001 |
| Pop 7 | 0.273 | 0.307 | 0.529 | 0.159 | 0.126 |  | 0.009 | 0.001 |
| Pop 8 | 0.293 | 0.328 | 0.523 | 0.173 | 0.160 | 0.053 |  | 0.001 |
| Pop 9 | 0.306 | 0.343 | 0.507 | 0.190 | 0.167 | 0.107 | 0.137 |  |
| B) Phi-values (all 871loci) | | | | | | | | |
|  | Pop 1 | Pop 2 | Pop 3 | Pop 4 | Pop 5+6 | Pop 7 | Pop 8 | Pop 9 |
| Pop 1 |  | 0.001 | 0.001 | 0.001 | 0.001 | 0.001 | 0.001 | 0.001 |
| Pop 2 | 0.129 |  | 0.001 | 0.001 | 0.001 | 0.001 | 0.001 | 0.001 |
| Pop 3 | 0.516 | 0.544 |  | 0.001 | 0.004 | 0.001 | 0.001 | 0.001 |
| Pop 4 | 0.325 | 0.363 | 0.510 |  | 0.001 | 0.001 | 0.001 | 0.001 |
| Pop 5+6 | 0.346 | 0.386 | 0.599 | 0.124 |  | 0.001 | 0.001 | 0.002 |
| Pop 7 | 0.296 | 0.328 | 0.553 | 0.156 | 0.121 |  | 0.008 | 0.001 |
| Pop 8 | 0.316 | 0.349 | 0.548 | 0.171 | 0.160 | 0.051 |  | 0.001 |
| Pop 9 | 0.329 | 0.363 | 0.539 | 0.189 | 0.169 | 0.107 | 0.138 |  |
| C) Phi-values (62 outlier loci) | | | | | | | | |
|  | Pop 1 | Pop 2 | Pop 3 | Pop 4 | Pop 5+6 | Pop 7 | Pop 8 | Pop 9 |
| Pop 1 |  | 0.001 | 0.001 | 0.001 | 0.001 | 0.001 | 0.001 | 0.001 |
| Pop 2 | 0.245 |  | 0.001 | 0.001 | 0.001 | 0.001 | 0.001 | 0.001 |
| Pop 3 | 0.688 | 0.666 |  | 0.001 | 0.001 | 0.001 | 0.001 | 0.001 |
| Pop 4 | 0.515 | 0.537 | 0.746 |  | 0.004 | 0.002 | 0.001 | 0.001 |
| Pop 5+6 | 0.549 | 0.552 | 0.810 | 0.077 |  | 0.253 | 0.001 | 0.001 |
| Pop 7 | 0.514 | 0.522 | 0.765 | 0.083 | 0.020 |  | 0.461 | 0.003 |
| Pop 8 | 0.542 | 0.550 | 0.782 | 0.125 | 0.170 | 0.000 |  | 0.001 |
| Pop 9 | 0.566 | 0.565 | 0.821 | 0.169 | 0.222 | 0.103 | 0.156 |  |
| D) Geographic distances | | | | | | | | |
|  | Pop 1 | Pop 2 | Pop 3 | Pop 4 | Pop 5+6 | Pop 7 | Pop 8 |  |
| Pop 1 |  |  |  |  |  |  |  |  |
| Pop 2 | 27.994 |  |  |  |  |  |  |  |
| Pop 3 | 144.338 | 135.374 |  |  |  |  |  |  |
| Pop 4 | 210.359 | 204.504 | 70.567 |  |  |  |  |  |
| Pop 5+6 | 231.542 | 225.577 | 91.149 | 21.188 |  |  |  |  |
| Pop 7 | 273.667 | 261.455 | 130.267 | 82.609 | 70.236 |  |  |  |
| Pop 8 | 275.442 | 263.156 | 132.097 | 84.478 | 71.971 | 1.912 |  |  |
| Pop 9 | 280.847 | 268.243 | 137.777 | 90.756 | 78.052 | 8.176 | 6.284 |  |
| E) Environmental dissimilarity | | | | | | | | |
|  | Pop 1 | Pop 2 | Pop 3 | Pop 4 | Pop 5+6 | Pop 7 | Pop 8 |  |
| Pop 1 |  |  |  |  |  |  |  |  |
| Pop 2 | 567.222 |  |  |  |  |  |  |  |
| Pop 3 | 880.338 | 1214.301 |  |  |  |  |  |  |
| Pop 4 | 734.600 | 752.663 | 608.163 |  |  |  |  |  |
| Pop 5+6 | 1087.471 | 1264.083 | 370.604 | 525.467 |  |  |  |  |
| Pop 7 | 1034.902 | 1267.865 | 257.367 | 560.393 | 131.625 |  |  |  |
| Pop 8 | 899.331 | 695.853 | 914.724 | 314.323 | 762.345 | 826.505 |  |  |
| Pop 9 | 816.270 | 895.361 | 511.829 | 178.412 | 391.931 | 422.018 | 422.848 |  |
| F) Latitudinal differentiation | | | | | | | | |
|  | Pop 1 | Pop 2 | Pop 3 | Pop 4 | Pop 5+6 | Pop 7 | Pop 8 |  |
| Pop 1 |  |  |  |  |  |  |  |  |
| Pop 2 | -0.002 |  |  |  |  |  |  |  |
| Pop 3 | 1.159 | 1.161 |  |  |  |  |  |  |
| Pop 4 | 1.798 | 1.800 | 0.639 |  |  |  |  |  |
| Pop 5+6 | 1.983 | 1.984 | 0.823 | 0.184 |  |  |  |  |
| Pop 7 | 2.012 | 2.014 | 0.853 | 0.214 | 0.029 |  |  |  |
| Pop 8 | 2.108 | 2.110 | 0.949 | 0.310 | 0.126 | 0.096 |  |  |
| Pop 9 | 2.133 | 2.135 | 0.974 | 0.335 | 0.151 | 0.121 | 0.025 |  |
| G) Bio4 (Temperature seasonality) | | | | | | | | |
|  | Pop 1 | Pop 2 | Pop 3 | Pop 4 | Pop 5+6 | Pop 7 | Pop 8 |  |
| Pop 1 |  |  |  |  |  |  |  |  |
| Pop 2 | 2.0763 |  |  |  |  |  |  |  |
| Pop 3 | 1.3927 | 1.2590 |  |  |  |  |  |  |
| Pop 4 | 1.6667 | 1.5476 | 1.5350 |  |  |  |  |  |
| Pop 5+6 | 1.6892 | 1.4959 | 1.4087 | 0.6111 |  |  |  |  |
| Pop 7 | 1.8828 | 1.7003 | 1.4461 | 0.5220 | 0.1587 |  |  |  |
| Pop 8 | 1.8498 | 1.6768 | 1.4952 | 0.7225 | 1.0625 | 0.0000 |  |  |
| Pop 9 | 1.8497 | 1.6472 | 1.6193 | 0.8895 | 1.3293 | 0.9626 | 1.1387 |  |
| H) Number of effective alleles | | | | | | | | |
|  | Pop 1 | Pop 2 | Pop 3 | Pop 4 | Pop 5+6 | Pop 7 | Pop 8 |  |
| Pop 1 |  |  |  |  |  |  |  |  |
| Pop 2 | 0.005 |  |  |  |  |  |  |  |
| Pop 3 | 0.32 | 0.315 |  |  |  |  |  |  |
| Pop 4 | 0.001 | -0.004 | -0.319 |  |  |  |  |  |
| Pop 5+6 | 0.038 | 0.033 | -0.282 | 0.037 |  |  |  |  |
| Pop 7 | 0.034 | 0.029 | -0.286 | 0.033 | -0.004 |  |  |  |
| Pop 8 | -0.002 | -0.007 | -0.322 | -0.003 | -0.04 | -0.036 |  |  |
| Pop 9 | -0.007 | -0.012 | -0.327 | -0.008 | -0.045 | -0.041 | -0.005 |  |
| I) Observed Heterozygosity | | | | | | | | |
|  | Pop 1 | Pop 2 | Pop 3 | Pop 4 | Pop 5+6 | Pop 7 | Pop 8 |  |
| Pop 1 |  |  |  |  |  |  |  |  |
| Pop 2 | 0.003 |  |  |  |  |  |  |  |
| Pop 3 | 0.167 | 0.164 |  |  |  |  |  |  |
| Pop 4 | -0.011 | -0.014 | -0.178 |  |  |  |  |  |
| Pop 5+6 | 0.005 | 0.002 | -0.162 | 0.016 |  |  |  |  |
| Pop 7 | 0.004 | 0.001 | -0.163 | 0.015 | -0.001 |  |  |  |
| Pop 8 | -0.023 | -0.026 | -0.19 | -0.012 | -0.028 | -0.027 |  |  |
| Pop 9 | -0.021 | -0.024 | -0.188 | -0.01 | -0.026 | -0.025 | 0.002 |  |
| J) Expected Heterozygosity | | | | | | | | |
|  | Pop 1 | Pop 2 | Pop 3 | Pop 4 | Pop 5+6 | Pop 7 | Pop 8 |  |
| Pop 1 |  |  |  |  |  |  |  |  |
| Pop 2 | 0.009 |  |  |  |  |  |  |  |
| Pop 3 | 0.202 | 0.193 |  |  |  |  |  |  |
| Pop 4 | -0.003 | -0.012 | -0.205 |  |  |  |  |  |
| Pop 5+6 | 0.024 | 0.015 | -0.178 | 0.027 |  |  |  |  |
| Pop 7 | 0.015 | 0.006 | -0.187 | 0.018 | -0.009 |  |  |  |
| Pop 8 | -0.004 | -0.013 | -0.206 | -0.001 | -0.028 | -0.019 |  |  |
| Pop 9 | -0.005 | -0.014 | -0.207 | -0.002 | -0.029 | -0.02 | -0.001 |  |
| K) Theta | | | | | | | | |
|  | Pop 1 | Pop 2 | Pop 3 | Pop 4 | Pop 5+6 | Pop 7 | Pop 8 |  |
| Pop 1 |  |  |  |  |  |  |  |  |
| Pop 2 | -0.01308 |  |  |  |  |  |  |  |
| Pop 3 | -0.00047 | 0.01261 |  |  |  |  |  |  |
| Pop 4 | 0.00197 | 0.01505 | 0.00244 |  |  |  |  |  |
| Pop 5+6 | -0.00133 | 0.01175 | -0.00086 | -0.0033 |  |  |  |  |
| Pop 7 | -0.00086 | 0.01222 | -0.00039 | -0.00283 | 0.00047 |  |  |  |
| Pop 8 | 0.00041 | 0.01349 | 0.00088 | -0.00156 | 0.00174 | 0.00127 |  |  |
| Pop 9 | -0.00255 | 0.01053 | -0.00208 | -0.00452 | -0.00122 | -0.00169 | -0.00296 |  |

Table S5. Correlation among variables. Values below and above the diagonal are *r* values and *P* values, respectively

|  | Geographic distance | Environmental dissimilarity | Latitudinal difference |
| --- | --- | --- | --- |
| Geographic distance |  | 0.026 | 0.001 |
| Environmental dissimilarity | 0.6118 |  | 0.013 |
| Latitudinal difference | 0.9695 | 0.6827 |  |

Table S6. AMOVA in *Dioon sonorense* and *D. vovidesii* using RAD-seq datasets consisting of neutral loci only, all loci, and outlier loci only.

|  | Source | **df** | **SS** | **MS** | **Est. Var.** | **%** |
| --- | --- | --- | --- | --- | --- | --- |
| 809 neutral loci | Among Species | 2 | 3299.660 | 3299.660 | 57.131 | 18% |
|  | Among Pops | 5 | 4542.877 | 757.146 | 53.423 | 17% |
|  | Within Pops | 81 | 16462.137 | 203.236 | 203.236 | 65% |
|  | Total | 88 | 24304.674 |  | 313.790 | 100% |
| All 871 loci | Among Species | 2 | 3889.181 | 3889.181 | 69.141 | 20% |
|  | Among Pops | 5 | 4990.682 | 831.780 | 59.449 | 17% |
|  | Within Pops | 81 | 17446.814 | 215.393 | 215.393 | 63% |
|  | Total | 88 | 26326.677 |  | 343.982 | 100% |
| 62 outlier loci | Among Species | 2 | 589.544 | 589.544 | 12.010 | 40% |
|  | Among Pops | 5 | 447.805 | 74.634 | 6.026 | 20% |
|  | Within Pops | 81 | 984.677 | 12.157 | 12.157 | 40% |
|  | Total | 88 | 2022.026 |  | 30.192 | 100% |

Table S7. Levels of genetic diversity of populations of *Dioon sonorense* (Pops 1–2) and *D. vovidesii* (Pops 3–9). Mean and standard error (SE) are annotated: *%P* = percentage of polymorphic loci. *A_e_* = Effective allele richness; *H_o_* = observed heterozygosity; *H_e_* = expected heterozygosity; *F* = fixation index; *I* = Shannon’s information index.

| Species | Pop |  | ***A_e_*** | ***H_o_*** | ***H_e_*** | ***F*** | ***I*** |
| --- | --- | --- | --- | --- | --- | --- | --- |
| *Dioon sonorense* | 1 | Mean | 1.403 | 0.240 | 0.246 | 0.003 | 0.378 |
|  |  | SE | 0.012 | 0.006 | 0.006 | 0.008 | 0.009 |
|  | 2 | Mean | 1.398 | 0.237 | 0.237 | -0.017 | 0.359 |
|  |  | SE | 0.013 | 0.007 | 0.007 | 0.008 | 0.009 |
|  | Overall | Mean | 1.414 | 0.238 | 0.254 | 0.041 | 0.390 |
|  |  | SE | 0.011 | 0.006 | 0.006 | 0.006 | 0.008 |
| *Dioon vovidesii* | 3 | Mean | 1.083 | 0.073 | 0.044 | -0.637 | 0.062 |
|  |  | SE | 0.009 | 0.008 | 0.005 | 0.009 | 0.007 |
|  | 4 | Mean | 1.402 | 0.251 | 0.249 | -0.023 | 0.386 |
|  |  | SE | 0.011 | 0.006 | 0.006 | 0.008 | 0.008 |
|  | 5+6 | Mean | 1.365 | 0.235 | 0.222 | -0.070 | 0.341 |
|  |  | SE | 0.012 | 0.008 | 0.006 | 0.011 | 0.009 |
|  | 7 | Mean | 1.369 | 0.236 | 0.231 | -0.045 | 0.359 |
|  |  | SE | 0.011 | 0.007 | 0.006 | 0.010 | 0.008 |
|  | 8 | Mean | 1.405 | 0.263 | 0.250 | -0.061 | 0.386 |
|  |  | SE | 0.011 | 0.007 | 0.006 | 0.009 | 0.008 |
|  | 9 | Mean | 1.410 | 0.261 | 0.251 | -0.050 | 0.388 |
|  |  | SE | 0.012 | 0.007 | 0.006 | 0.009 | 0.008 |
|  | Overall | Mean | 1.406 | 0.232 | 0.266 | 0.102 | 0.423 |
|  |  | SE | 0.009 | 0.004 | 0.005 | 0.007 | 0.006 |

Table S8. Theta (*Θ*) values and levels of gene flow between species estimated in MIGRATE-N v3.6 (Beerli, 2005) Theta is an estimate of population size estimated as *Θ* = 4*N_e_µ*, where *N_e_* is the historical effective population size and µ is mutation rate. Mean pairwise mutation-scaled migration rates (*M*) and distribution of posterior probabilities at 2.5% and 97.5% percentiles. Numbers of migrants (*Nm*) per generation were estimated via *Θ_a_M_a_*_->_*_b_* = 4*Nm_a_*.

| **Parameter** | **Mean** | **2.5–97.5%** | ***Nm*** |
| --- | --- | --- | --- |
| *Θ* _Dson_ | 0.01011 | 0.00583—0.01417 |  |
| *Θ* _Dvov_ | 0.01669 | 0.01250—0.02083 |  |
| *M* _Dvov to Dson_ | 56.7 | 22.7—89.3 | 0.237 |
| *M* _Dson to Dvov_ | 48.3 | 13.3—81.3 | 0.122 |

Table S9. Parameters theta (*Θ*) and posterior probabilities at 2.5% and 97.5% percentiles of populations estimated in MIGRATE-N v3.6 (Beerli, 2005). Effective population size (Ne) was estimated via *Θ* = 4*N_e_µ,* considering µ = 6.70325 × 10^-10^ substitutions per site per year, as estimated by De La Torre et al. (2017), and generation times of 100 years as estimated by Vovides (1990).

|  | ***Θ* (Mean )** | **2.5–97.5% percentiles** | ***Ne*** |
| --- | --- | --- | --- |
| Pop1 | 0.00922 | 0.00500—0.01333 | 34386 |
| Pop2 | 0.02230 | 0.01800—0.02767 | 83169 |
| Pop3 | 0.00969 | 0.00533—0.01433 | 36139 |
| Pop4 | 0.00725 | 0.00300—0.01133 | 27039 |
| Pop5+6 | 0.01055 | 0.00633—0.01467 | 39347 |
| Pop7 | 0.01008 | 0.00583—0.01417 | 37594 |
| Pop8 | 0.00881 | 0.00450—0.01283 | 32857 |
| Pop9 | 0.01177 | 0.00750—0.01583 | 43897 |

Table S10. Theta (*Θ*) values and levels of gene flow among lineages estimated in MIGRATE-N v3.6 (Beerli, 2005) Theta is an estimate of population size estimated as *Θ* = 4*N_e_µ*, where *N_e_* is the historical effective population size and µ is mutation rate. Mean pairwise mutation-scaled migration rates (*M*) and distribution of posterior probabilities at 2.5% and 97.5% percentiles. Numbers of migrants (*Nm*) per generation were estimated via *Θ_a_M_a_*_->_*_b_* = 4*Nm_a_*.

| **Parameter** | **Mean** | **2.5–97.5%n percentiles** | ***Nm*** |
| --- | --- | --- | --- |
| *Θ* _Dson_ | 0.00994 | 0.0055—0.0145 |  |
| *Θ* _Dvov-S_ | 0.00751 | 0.00317–0.01167 |  |
| *Θ* _Dvov-N_ | 0.01398 | 0.00967—0.18 |  |
| *M* _Dvov-S to Dson_ | 36.9 | 2.7—69.3 | 0.069 |
| *M* _Dvov-N to Dson_ | 63.4 | 28—97.3 | 0.222 |
| *M* _Dson to Dvov-S_ | 173.1 | 129.3—218.7 | 0.430 |
| *M* _Dvov-N toDvov-S_ | 114.5 | 80—148 | 0.400 |
| *M* _Dson to Dvov-N_ | 123 | 72—168 | 0.231 |
| *M* _Dvov-S toDvov-N_ | 44.1 | 9.3—77.3 | 0.083 |

Table S11. Estimation of Mean pairwise mutation-scaled migration rates (*M*) and distribution of posterior probabilities at 2.5% and 97.5% percentiles obtained with MIGRATE-N v3.6 (Beerli, 2005). Numbers of migrants (*Nm*) over generations were estimated via *Θ_a_M_a_*_->_*_b_* = 4*Nm_a_*.

| ***Parameter*** | **Mean** | **2.5–97.5%** | ***Nm*** |  | ***Parameter*** | ***Mean*** | ***2.5–97.5%*** | ***Nm*** |
| --- | --- | --- | --- | --- | --- | --- | --- | --- |
| *M* _2 to 1_ | 38 | 4—70.7 | 0.212 |  | *M* _1 to 5+6_ | 39.2 | 5.3—72 | 0.090 |
| *M* _3 to 1_ | 29.4 | 0—60 | 0.071 |  | *M* _2 to 5+6_ | 41.0 | 6.7—73.3 | 0.229 |
| *M* _4 to 1_ | 36.7 | 2.7—69.3 | 0.067 |  | *M* _3 to 5+6_ | 32.7 | 0–64 | 0.079 |
| *M* _5+6 to 1_ | 27.6 | 0—57.3 | 0.073 |  | *M* _4 to 5+6_ | 52.1 | 18.7—85.3 | 0.094 |
| *M* _7 to 1_ | 28.7 | 0–58.7 | 0.072 |  | *M* _7 to 5+6_ | 34.0 | 1.3—65.3 | 0.086 |
| *M* _8 to 1_ | 34.8 | 1.3—66.7 | 0.077 |  | *M* _8 to 5+6_ | 40.1 | 6.7—73.3 | 0.088 |
| *M* _9 to 1_ | 34.5 | 1.3—66.7 | 0.102 |  | *M* _9 to 5+6_ | 35.3 | 1.3—66.7 | 0.104 |
| *M* _1 to 2_ | 38 | 4—70.7 | 0.088 |  | *M* _1 to 7_ | 49.5 | 14.7—82.7 | 0.114 |
| *M* _3 to 2_ | 26 | 0—57.3 | 0.063 |  | *M* _2 to 7_ | 35.4 | 2.7—68 | 0.197 |
| *M* _4 to 2_ | 38 | 4–61.3 | 0.069 |  | *M* _3 to 7_ | 25.9 | 0—56 | 0.063 |
| *M* _5+6 to 2_ | 27.3 | 0—57.3 | 0.072 |  | *M* _4 to 7_ | 39.3 | 5.3—72 | 0.071 |
| *M* _7 to 2_ | 31.2 | 0—61.3 | 0.079 |  | *M* _5+6 to 7_ | 42.7 | 9.3—76 | 0.113 |
| *M* _8 to 2_ | 27.4 | 0—57.3 | 0.060 |  | *M* _8 to 7_ | 35.5 | 2.7—68 | 0.078 |
| *M* _9 to 2_ | 36.5 | 2.7—69.3 | 0.107 |  | *M* _9 to 7_ | 26.4 | 0—57.3 | 0.078 |
| *M* _1 to 3_ | 37.8 | 2.7—72 | 0.087 |  | *M* _1 to 8_ | 35.7 | 2.7—68 | 0.082 |
| *M* _2 to 3_ | 47.6 | 13.3—80 | 0.265 |  | *M* _2 to 8_ | 46.7 | 12—80 | 0.260 |
| *M* _4 to 3_ | 35.3 | 2.7—68 | 0.064 |  | *M* _3 to 8_ | 28.2 | 0—58.7 | 0.068 |
| *M* _5+6 to 3_ | 31.5 | 0—62.7 | 0.083 |  | *M* _4 to 8_ | 46.2 | 12—78.7 | 0.084 |
| *M* _7 to 3_ | 36.3 | 2.7—69.3 | 0.091 |  | *M* _5+6 to 8_ | 36.0 | 2.7—68 | 0.095 |
| *M* _8 to 3_ | 35.4 | 2.7—68 | 0.078 |  | *M* _7 to 8_ | 37.0 | 2.7—69.3 | 0.093 |
| *M* _9 to 3_ | 44.3 | 10.7—77.3 | 0.130 |  | *M* _9 to 8_ | 38.0 | 4—70.7 | 0.112 |
| *M* _1 to 4_ | 42.9 | 9.3–76 | 0.099 |  | *M* _1 to 9_ | 48.5 | 14.7—81.3 | 0.112 |
| *M* _2 to 4_ | 41.8 | 8—74.7 | 0.233 |  | *M* _2 to 9_ | 36.2 | 1.3—69.3 | 0.202 |
| *M* _3 to 4_ | 28.6 | 0-58.7 | 0.069 |  | *M* _3 to 9_ | 30.0 | 0—60 | 0.073 |
| *M* _5+6 to 4_ | 31.8 | 0–62.7 | 0.084 |  | *M* _4 to 9_ | 54.3 | 20—88 | 0.098 |
| *M* _7 to 4_ | 31 | 0–61.3 | 0.078 |  | *M* _5+6 to 9_ | 36.9 | 2.7—69.3 | 0.097 |
| *M* _8 to 4_ | 33.8 | 1.3—65.3 | 0.074 |  | *M* _7 to 9_ | 36.7 | 2.7—69.3 | 0.092 |
| *M* _9 to 4_ | 35.4 | 2.7—68 | 0.104 |  | *M* _8 to 9_ | 36.8 | 2.7—69.3 | 0.081 |

Table S12. Posterior parameter estimates for the best-supported scenarios (Scenario 2) in Approximate Bayesian Computation. Estimates are based on 1% of simulated datasets closest to the observed values. Distribution of posterior probabilities at 2.5% and 97.5% percentiles are indicated. Parameters correspond to those indicated in Fig. 2 and Fig. S9. Mean relative bias and relative square root error (median) of parameters of Scenario 2 are indicated.

| Parameter | Mean | Median | Mode | 2.5–97.5% quantiles | Mean relative bias (medians) | Relative square root error (medians) |
| --- | --- | --- | --- | --- | --- | --- |
| *N1* | 3E+04 | 2.93E+04 | 2.67E+04 | 1.75E+04 – 4.6E+04 | 0.448 (0.375) | 0.655 (0.581) |
| *N2* | 1.48E+03 | 1.34E+03 | 1.29E+03 | 6.47E+02 – 3.08E+03 | -0.043 (13.824) | 0.220 (14.586) |
| *N3* | 6.1E+04 | 6.11E+04 | 6.04E+04 | 4.37E+04 – 7.71E+04 | -0.106 (-0.138) | 0.162 (0.180) |
| *t1* | 7.08E+03 | 6.93E+03 | 6.82E+03 | 3.95E+03 – 1.11E+04 | 0.087 (1.124) | 0.247 (1.289) |
| *Na* | 3.66E+04 | 3.71E+04 | 3.55E+04 | 7.89E+03 – 6.46E+04 | 0.246 (-0.048) | 1.653 (1.405) |
| *Nc* | 9.25E+03 | 8.82E+03 | 8.62E+03 | 4.6E+03 – 1.69E+04 | 0.468 (1.842) | 0.654 (2.135) |
| *t2* | 8.46E+03 | 8.29E+03 | 7.78E+03 | 5.08E+03 – 1.27E+04 | 0.184 (3.519) | 0.325 (3.620) |

Table 13. Differentiation tests of climate variation among populations. ANOVA Welch’s F and Kruskal-Wallis’ H (χ2) values are annotated. Highlighted cells indicate variables with significant differentiation between the two species. * = *P* < 0.05, ** = *P* < 0.005, *** = *P* < 0.001.

| Bioclimate variables | Abbreviation | *F* | *H* (χ^2^) | Used in PCA and ENMs? |
| --- | --- | --- | --- | --- |
| Annual mean temperature | bio1 | 0.0083 | 0.1429 | No |
| Mean diurnal range temperature | bio2 | 1.051 | 0.317 | Yes |
| Isothermality | bio3 | 191.6*** | 15.75*** | Yes |
| Temperature seasonality | bio4 | 62.97*** | 15.75*** | Yes |
| Max temperature of the warmest month | bio5 | 0.9016 | 1.016 | Yes |
| Min temperature of the coldest month | bio6 | 5.695* | 3.571 | Yes |
| Temperature annual range | bio7 | 6.884* | 4.723* | Yes |
| Mean temperature of wettest quarter | bio8 | 2.146 | 1.668 | No |
| Mean temperature of driest quarter | bio9 | 0.0032 | 0.0158 | Yes |
| Mean temperature of the warmest quarter | bio10 | 2.177 | 1.834 | No |
| Mean temperature of the coldest quarter | bio11 | 3.42 | 2.286 | No |
| Annual precipitation | bio12 | 31.85*** | 12.89*** | Yes |
| Precipitation of wettest month | bio13 | 31.85*** | 12.89*** | No |
| Precipitation of driest month | bio14 | 0.6632 | 0.0357 | Yes |
| Precipitation seasonality | bio15 | 29.63*** | 12.67*** | Yes |
| Precipitation of wettest quarter | bio16 | 58.72*** | 15.75*** | No |
| Precipitation of driest quarter | bio17 | 3.23 | 3.571 | No |
| Precipitation of the warmest quarter | bio18 | 33.98*** | 14.29*** | Yes |
| Precipitation of the coldest quarter | bio19 | 2.271 | 2.286 | Yes |
| Altitude | alt | 0.0001 | 0.0158 | No |

Table S14: Bioclimatic variables and their contribution to the construction of the ENMs of *D. sonorense* and *D. vovidesii*.

|  | ***D. sonorense*** | | ***D. vovidesii*** | |
| --- | --- | --- | --- | --- |
| **Variable** | **Percent contribution** | **Permutation importance** | **Percent contribution** | **Permutation importance** |
| bio2 | **13.6** | **19** | 0.6 | 0.3 |
| bio3 | 1.5 | **12.1** | 5.3 | **36.5** |
| bio4 | **17.1** | **23.3** | **25.7** | 5.8 |
| bio5 | **37.5** | 6.4 | **18.1** | 7.4 |
| bio6 | 0.6 | 2.6 | **15.2** | **8.5** |
| bio7 | 0 | 0.9 | 0 | 0 |
| bio9 | 0.1 | 0 | 0.7 | 0.1 |
| bio12 | 4.3 | 10.3 | 0.3 | 1.6 |
| bio14 | 2.4 | 1.9 | 6.3 | **11.6** |
| bio15 | 8.2 | 4.2 | 0.4 | 0.2 |
| bio18 | 1.5 | 5.6 | 0.8 | 1.1 |
| bio19 | **13.2** | **13.8** | **26.5** | **27** |

Table S15: Bioclimatic variables and their contribution to the construction of the Principal Components PC1 and PC2. Bold values indicate the highest loading score for each principal component.

| **Variable** | **PC1** | **PC2** |
| --- | --- | --- |
| bio2 | 0.0043 | 0.1013 |
| bio3 | -0.0039 | 0.0124 |
| bio4 | **0.9855** | 0.1489 |
| bio5 | 0.0273 | 0.1450 |
| bio6 | -0.0040 | 0.0282 |
| bio7 | 0.0312 | 0.1167 |
| bio9 | 0.0130 | 0.1250 |
| bio12 | -0.1424 | **0.7892** |
| bio14 | -0.0001 | 0.0109 |
| bio15 | -0.0059 | 0.0009 |
| bio18 | -0.0809 | 0.5413 |
| bio19 | -0.0007 | 0.0194 |

Table S16. Multiple linear regression models including genetic differentiation among population estimated as phi-values for the (A) “neutral”, (B) “all loci” and (C) “outlier” datasets) as the dependent variables and geographic distances (Geo), environmental dissimilarity (Env), and latitudinal differentiation (Lat) as independent variables. Models were evaluated using the Akaike Information Criterion corrected for small samples (AICc). Highlighted rows indicate the best models according to the AICc values.

| **A) Neutral loci dataset** | | | | | | |
| --- | --- | --- | --- | --- | --- | --- |
|  |  | **Intercept** | **SE** | **t-value** | **Pr(>\|t\|)** | **AICc** |
| Model1 | Geo + Env + Lat | 3.664E-05 | 1.704E-01 | 0.000 | 0.9998 | 82.09042 |
| Model2 | Geo + Env | 0.0002205 | 0.1795680 | 0.001 | 0.999 | 83.16494 |
| Model3 | Geo + Lat | 6.647E-05 | 1.684E-01 | 0.000 | 0.9997 | 79.58456 |
| Model4 | Env + Lat | 0.0001574 | 0.1732476 | 0.001 | 0.999 | 81.15835 |
| Model5 | Geo | 0.0002197 | 0.1763301 | 0.001 | 0.9990 | 80.505 |
| Model6 | Env | 0.0002281 | 0.1846887 | 0.001 | 0.999 | 83.09857 |
| Model7 | Lat | 0.0001636 | 0.1701385 | 0.001 | 0.9992 | 78.50327 |
| Model8 | Geo + PC1 + Lat | 3.772E-05 | 1.69E-01 | 0.000 | 0.9998 | 81.5997 |
| Model9 | Geo + PC1 | 0.0002186 | 0.1795468 | 0.001 | 0.9990 | 83.15831 |
| Model10 | PC1 + Lat | 0.0001394 | 0.1710102 | 0.001 | 0.9994 | 80.4304 |
| Model11 | PC1 | 0.0002285 | 0.1860699 | 0.001 | 0.999 | 83.51582 |
| Model12 | Geo + bio4 + Lat | 3.832E-05 | 1.699E-01 | 0.000 | 0.9998 | 81.90739 |
| Model13 | Geo + bio4 | 0.0002196 | 0.1798199 | 0.001 | 0.999 | 83.24343 |
| Model14 | bio4 + Lat | 0.0001447 | 0.1721378 | 0.001 | 0.9993 | 80.79844 |
| Model15 | bio4 | 0.0002285 | 0.1841601 | 0.001 | 0.999 | 82.93806 |
| Model16 | Lat*Env | 0.31976 | 0.22806 | 1.402 | 0.1737 | 79.78598 |
| Model17 | Lat:Env | 0.2220 | 0.2586 | 0.859 | 0.398 | 83.85651 |
| Model18 | Lat*Geo | 1.0504 | 0.1599 | 6.570 | 8.54E-07 | 46.42471 |
| Model19 | Lat:Geo | 0.7454 | 0.2756 | 2.704 | 0.01191 | 75.50869 |
| Model20 | Geo*Env | 0.32640 | 0.23431 | 1.393 | 0.1764 | 81.77279 |
| Model21 | Geo:Env | 0.1818 | 0.2546 | 0.714 | 0.482 | 84.28483 |
| Model22 | Lat*Geo + Env | 1.04415 | 0.16256 | 6.423 | 1.49E-06 | 49.30764 |
| Model23 | Lat:Geo + Env | 0.9136 | 0.2436 | 3.751 | 0.000936 | 68.84625 |
| Model24 | Lat*Env + Geo | 0.2768 | 0.2355 | 1.175 | 0.252 | 82.25156 |
| Model25 | Lat:Env + Geo | 0.3434 | 0.2330 | 1.474 | 0.15302 | 78.70876 |
| Model26 | Geo*Env + Lat | 0.2889 | 0.2257 | 1.280 | 0.2133 | 81.50113 |
| Model27 | Geo:Env + Lat | 0.3215 | 0.2193 | 1.466 | 0.15515 | 76.55101 |
| Model28 | Lat*Geo + PC1 | 1.0393 | 0.1590 | 6.537 | 1.14E-06 | 48.0933 |
| Model29 | Lat:Geo + PC1 | 0.9235 | 0.2470 | 3.738 | 0.000967 | 69.40299 |
| Model30 | Lat*Geo + bio4 | 1.0474 | 0.1575 | 6.648 | 8.79E-07 | 47.6756 |
| Model31 | Lat:Geo + bio4 | 0.9089 | 0.2424 | 3.749 | 0.000941 | 68.67902 |
| Model32 | Lat*Geo + Lat*Env | 1.09630 | 0.17386 | 6.306 | 2.4E-06 | 51.94549 |
| Model33 | Lat:Geo + Lat:Env | 0.7813 | 0.2940 | 2.658 | 0.01351 | 78.06613 |
| Model34 | Lat*Geo + Lat*pc1 | 1.1164 | 0.1601 | 6.971 | 5.36E-07 | 48.3927 |
| Model35 | Lat:Geo + Lat:pc1 | 0.77144 | 0.29087 | 2.652 | 0.0137 | 78.12064 |
| Model36 | Lat*Geo + Lat*bio4 | 1.1197 | 0.1612 | 6.948 | 5.64E-07 | 48.60813 |
| Model37 | Lat:Geo + Lat:bio4 | 0.7726 | 0.2924 | 2.643 | 0.0140 | 78.1262 |
| **B) All loci dataset** | | | | | | |
| Model1 | Geo + Env + Lat | -0.0001507 | 0.1678893 | -0.001 | 0.9993 | 81.24411 |
| Model2 | Geo + Env | 3.452E-05 | 1.774E-01 | 0.000 | 1.000 | 82.49265 |
| Model3 | Geo + Lat | -0.000121 | 0.165951 | -0.001 | 0.999 | 78.74872 |
| Model4 | Env + Lat | -3.128E-05 | 1.707E-01 | 0.000 | 0.9999 | 80.32765 |
| Model5 | Geo | 3.376E-05 | 1.743E-01 | 0.000 | 0.9998 | 79.84091 |
| Model6 | Env | 0.0000426 | 0.1837020 | 0.000 | 1.00 | 82.79859 |
| Model7 | Lat | -2.508E-05 | 1.676E-01 | 0.000 | 0.99988 | 77.67604 |
| Model8 | Geo + PC1 + Lat | -0.0001507 | 0.1661633 | -0.001 | 0.9993 | 80.66541 |
| Model9 | Geo + PC1 | 3.249E-05 | 1.774E-01 | 0.000 | 0.9999 | 82.47771 |
| Model10 | PC1 + Lat | -0.0000501 | 0.1682428 | 0.000 | 0.9998 | 79.51676 |
| Model11 | PC1 | 0.000043 | 0.185251 | 0.000 | 1.000 | 83.26886 |
| Model12 | Geo + bio4 + Lat | -0.0001515 | 0.1669454 | -0.001 | 0.9993 | 80.92837 |
| Model13 | Geo + bio4 | 3.343E-05 | 1.777E-01 | 0.000 | 1.000 | 82.57351 |
| Model14 | bio4 + Lat | -4.583E-05 | 1.693E-01 | 0.000 | 0.9998 | 79.85858 |
| Model15 | bio4 | 0.000043 | 0.183443 | 0.000 | 1.000 | 82.71958 |
| Model16 | Lat*Env | 0.31944 | 0.22415 | 1.425 | 0.1670 | 78.81679 |
| Model17 | Lat:Env | 0.2176 | 0.2588 | 0.841 | 0.408 | 83.89585 |
| Model18 | Lat*Geo | 1.0228 | 0.1622 | 6.307 | 1.61E-06 | 47.2178 |
| Model19 | Lat:Geo | 0.7106 | 0.2808 | 2.531 | 0.01777 | 76.54338 |
| Model20 | Geo*Env | 0.32203 | 0.23155 | 1.391 | 0.1771 | 81.10913 |
| Model21 | Geo:Env | 0.1715 | 0.2551 | 0.672 | 0.507 | 84.39285 |
| Model22 | Lat*Geo + Env | 1.01646 | 0.16486 | 6.166 | 2.73E-06 | 50.09605 |
| Model23 | Lat:Geo + Env | 0.8821 | 0.2480 | 3.558 | 0.00153 | 69.85267 |
| Model24 | Lat*Env + Geo | 0.2774 | 0.2315 | 1.198 | 0.243 | 81.28652 |
| Model25 | Lat:Env + Geo | 0.3446 | 0.2296 | 1.501 | 0.14592 | 77.88524 |
| Model26 | Geo*Env + Lat | 0.2841 | 0.2223 | 1.278 | 0.2141 | 80.66231 |
| Model27 | Geo:Env + Lat | 0.3164 | 0.2161 | 1.464 | 0.15565 | 75.72954 |
| Model28 | Lat*Geo + PC1 | 1.0111 | 0.1608 | 6.288 | 2.05E-06 | 48.72596 |
| Model29 | Lat:Geo + PC1 | 0.8915 | 0.2519 | 3.539 | 0.001601 | 70.49791 |
| Model30 | Lat*Geo + bio4 | 1.0195 | 0.1588 | 6.421 | 1.49E-06 | 48.11253 |
| Model31 | Lat:Geo + bio4 | 0.8756 | 0.2479 | 3.532 | 0.001628 | 69.92474 |
| Model32 | Lat*Geo + Lat*Env | 1.07250 | 0.17595 | 6.095 | 3.9E-06 | 52.61351 |
| Model33 | Lat:Geo + Lat:Env | 0.7485 | 0.2994 | 2.500 | 0.0193 | 79.08759 |
| Model34 | Lat*Geo + Lat*pc1 | 1.0881 | 0.1622 | 6.708 | 9.63E-07 | 49.10757 |
| Model35 | Lat:Geo + Lat:pc1 | 0.73501 | 0.29640 | 2.480 | 0.0202 | 79.17489 |
| Model36 | Lat*Geo + Lat*bio4 | 1.0920 | 0.1625 | 6.719 | 9.39E-07 | 49.07782 |
| Model37 | Lat:Geo + Lat:bio4 | 0.73638 | 0.29791 | 2.472 | 0.0206 | 79.17757 |
| **C) Outlier loci dataset** | | | | | | |
| Model1 | Geo + Env + Lat | -0.0001513 | 0.1490932 | -0.001 | 0.999 | 74.59505 |
| Model2 | Geo + Env | 4.156E-05 | 1.617E-01 | 0.000 | 0.9998 | 77.28271 |
| Model3 | Geo + Lat | -0.0001222 | 0.1476593 | -0.001 | 0.9993 | 72.20875 |
| Model4 | Env + Lat | -4.091E-05 | 1.52E-01 | 0.000 | 0.99979 | 73.8467 |
| Model5 | Geo | 4.066E-05 | 1.589E-01 | 0.000 | 0.99980 | 74.69221 |
| Model6 | Env | 5.239E-05 | 1.771E-01 | 0.000 | 0.9998 | 80.74993 |
| Model7 | Lat | -3.433E-05 | 1.494E-01 | 0.000 | 0.999818 | 71.23202 |
| Model8 | Geo + PC1 + Lat | -0.0001603 | 0.1447039 | -0.001 | 0.9991 | 72.92164 |
| Model9 | Geo + PC1 | 3.849E-05 | 1.611E-01 | 0.000 | 0.9998 | 77.07053 |
| Model10 | PC1 + Lat | -6.696E-05 | 1.471E-01 | 0.000 | 0.999641 | 72.01175 |
| Model11 | PC1 | 5.291E-05 | 1.805E-01 | 0.000 | 0.9998 | 81.82004 |
| Model12 | Geo + bio4 + Lat | -0.0001709 | 0.1436352 | -0.001 | 0.9991 | 72.50653 |
| Model13 | Geo + bio4 | 3.861E-05 | 1.612E-01 | 0.000 | 0.9998 | 77.12072 |
| Model14 | bio4 + Lat | -6.974E-05 | 1.468E-01 | 0.000 | 0.999625 | 71.87409 |
| Model15 | bio4 | 0.0000529 | 0.1798443 | 0.000 | 0.9998 | 81.61008 |
| Model16 | Lat*Env | 0.31442 | 0.19577 | 1.606 | 0.121336 | 71.23491 |
| Model17 | Lat:Env | 0.1878 | 0.2607 | 0.720 | 0.478 | 84.29832 |
| Model18 | Lat*Geo | 0.8069 | 0.1779 | 4.536 | 0.000135 | 52.39949 |
| Model19 | Lat:Geo | 0.4558 | 0.3102 | 1.470 | 0.1537 | 82.11774 |
| Model20 | Geo*Env | 0.28858 | 0.21157 | 1.364 | 0.18522 | 76.05405 |
| Model21 | Geo:Env | 0.1026 | 0.2582 | 0.397 | 0.694 | 85.07327 |
| Model22 | Lat*Geo + Env | 0.79932 | 0.18062 | 4.426 | 0.000195 | 55.20706 |
| Model23 | Lat:Geo + Env | 0.28858 | 0.21157 | 1.364 | 0.18522 | 75.58756 |
| Model24 | Lat*Env + Geo | 0.2775 | 0.2022 | 1.373 | 0.1830 | 73.69875 |
| Model25 | Lat:Env + Geo | 0.3488 | 0.2050 | 1.702 | 0.101255 | 71.53015 |
| Model26 | Geo*Env + Lat | 0.2482 | 0.1979 | 1.254 | 0.2224 | 74.14573 |
| Model27 | Geo:Env + Lat | 0.2787 | 0.1931 | 1.444 | 0.1612 | 69.40402 |
| Model28 | Lat*Geo + PC1 | 0.7892 | 0.1712 | 4.610 | 0.000123 | 52.23737 |
| Model29 | Lat:Geo + PC1 | 0.6457 | 0.2833 | 2.280 | 0.03143 | 77.06159 |
| Model30 | Lat*Geo + bio4 | 0.8014 | 0.1636 | 4.898 | 6.01E-05 | 49.79075 |
| Model31 | Lat:Geo + bio4 | 0.6226 | 0.2825 | 2.204 | 0.03698 | 77.24926 |
| Model32 | Lat*Geo + Lat*Env | 0.88249 | 0.18957 | 4.655 | 0.000122 | 56.78741 |
| Model33 | Lat:Geo + Lat:Env | 0.5090 | 0.3300 | 1.542 | 0.136 | 84.54106 |
| Model34 | Lat*Geo + Lat*pc1 | 0.8581 | 0.1757 | 4.883 | 7E-05 | 53.59869 |
| Model35 | Lat:Geo + Lat:pc1 | 0.4630 | 0.3280 | 1.412 | 0.170 | 84.84932 |
| Model36 | Lat*Geo + Lat*bio4 | 0.8651 | 0.1697 | 5.098 | 4.15E-05 | 51.49661 |
| Model37 | Lat:Geo + Lat:bio4 | 0.46380 | 0.32966 | 1.407 | 0.172 | 84.84867 |


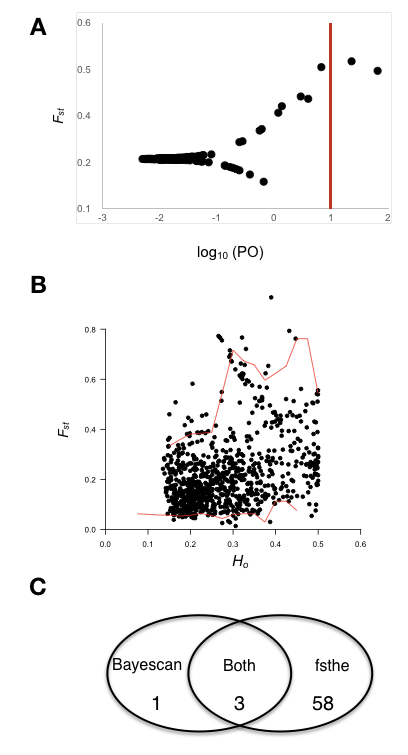


Fig. S1. Outlier detection test results on 871 loci of *Dioon sonorense* and *D. vovidesii*. (A) Plot showing the relationship between *F_st_* values and log_10_ prior odd (PO) estimated in BayeScan (Foll and Gaggiotti, 2008). Loci with log10 (PO) values above 1 were considered as outliers; two loci with log10 (PO) > 1000 were excluded. (B) Plot showing the relationship between heterozygosity and F_st_ values estimated in the R package fsthet (Flanagan and Jones, 2017). Loci below and above confidence intervals 95% (red lines) were considered as outliers. (C) Venn diagram showing the number of outlier loci identified by the two methods used in this study.


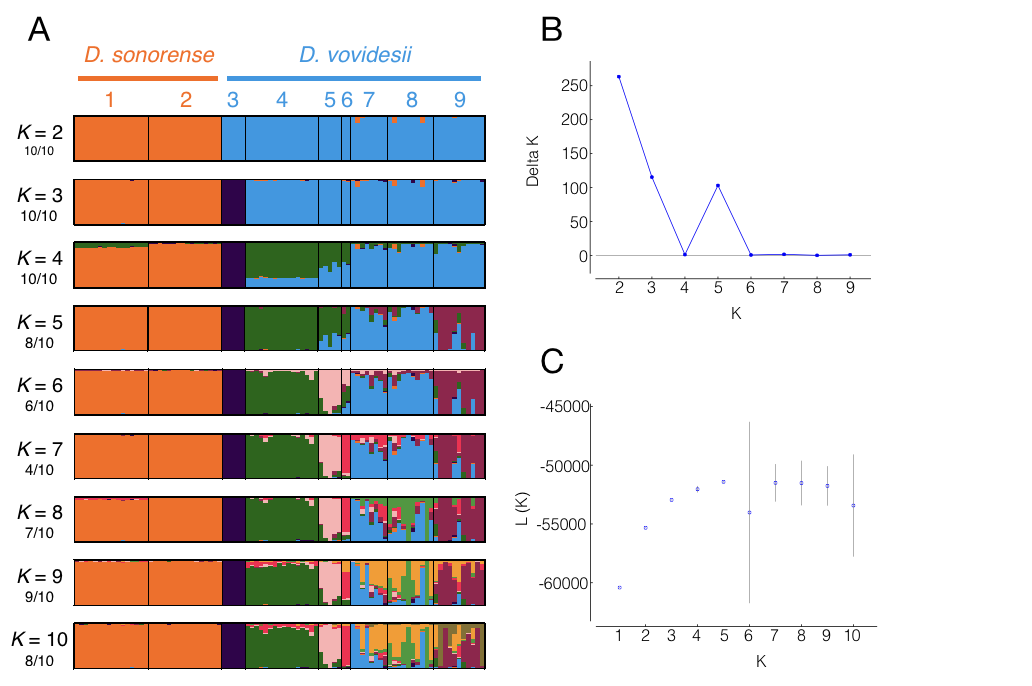


Fig. S2. Genetic clustering of populations of *Dioon sonorense* and *D. vovidesii*. (A) Genetic clusters in STRUCTURE (Pritchard et al., 2010) were estimated from the variation of 809 neutral loci from nine populations. Clusters (*K*) = 2–10 are shown. The proportion of major clustering calculated for each *K* value in Clumpak (Kopelman et al., 2015) is indicated. (B) Plot of Delta *K* values and (C) mean likelihood L(*K*) and variance per value from STRUCTURE calculated in with the method of Evanno *et al.* (2005) in Structure Harvester (Earl and vonHoldt *et al*. 2012) are displayed.


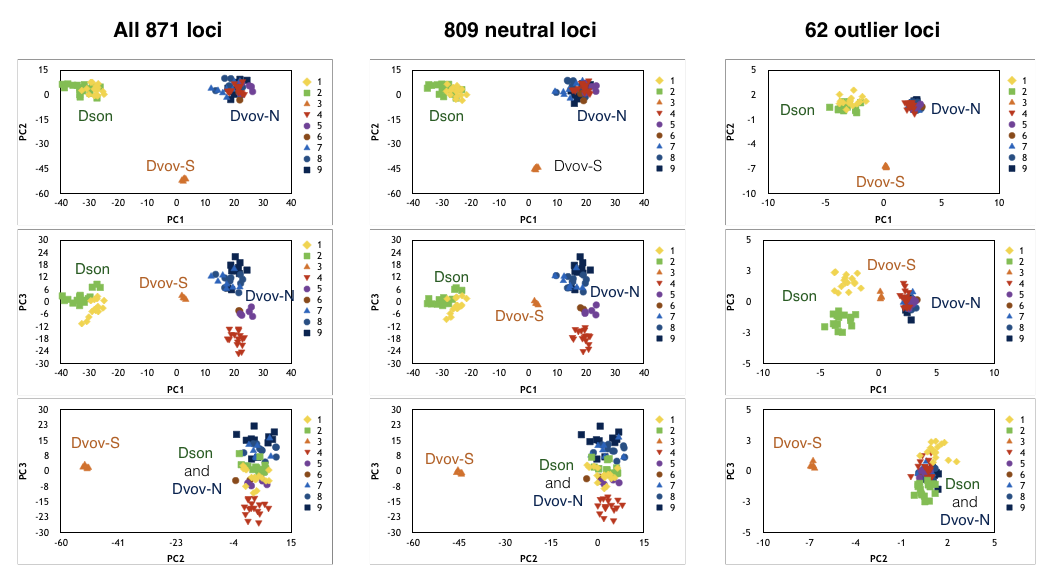


Fig. S3. Population group revealed by different combinations of principal coordinates (PCo1-3) showing the dispersion of genetic variation of three datasets: “all loci dataset” (871 loci), “neutral dataset” (809 loci), and “outlier loci dataset” (62 loci).


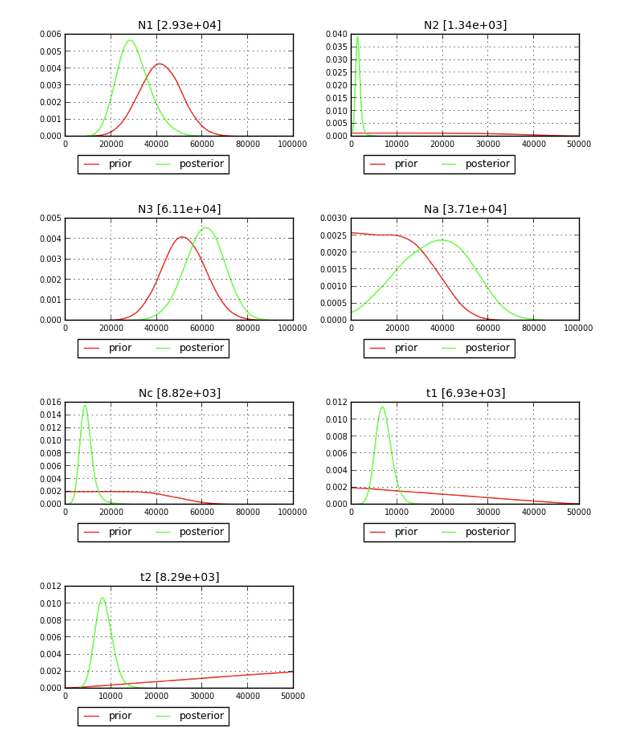


Fig S4. Estimation of demographic parameters in the Approximate Bayesian Computation. Plots represent the distribution of prior and posterior probability values estimated for the seven parameters of the most probable model: Scenario 2. All parameters reached convergence (one peak). Names of parameters correspond to those indicated in Fig. 2, Table S12.
